# GM2 ganglioside accumulation causes neuroinflammation and behavioral alterations in a mouse model of early onset Tay-Sachs disease

**DOI:** 10.1186/s12974-020-01947-6

**Published:** 2020-09-20

**Authors:** Seçil Akyıldız Demir, Zehra Kevser Timur, Nurselin Ateş, Luis Alarcón Martínez, Volkan Seyrantepe

**Affiliations:** 1 Department of Molecular Biology and Genetics, Izmir Institute of Technology, 35430 Izmir, Turkey; 2 Institute of Neurological Science and Psychiatry, Hacettepe University, Sihhiye, 06100 Ankara, Turkey

**Keywords:** Tay-Sachs disease, GM2, Mouse model, Neuroinflammation, Behavior

## Abstract

**Background:**

Tay-Sachs disease (TSD), a type of GM2-gangliosidosis, is a progressive neurodegenerative lysosomal storage disorder caused by mutations in the *α* subunit of the lysosomal β-hexosaminidase enzyme. This disease is characterized by excessive accumulation of GM2 ganglioside, predominantly in the central nervous system. Although Tay-Sachs patients appear normal at birth, the progressive accumulation of undegraded GM2 gangliosides in neurons leads to death. Recently, an early onset Tay-Sachs disease mouse model, with genotype *Hexa−*/*−Neu3−*/*−*, was generated. Progressive accumulation of GM2 led to premature death of the double KO mice. Importantly, this double-deficient mouse model displays typical features of Tay-Sachs patients, such as cytoplasmic vacuolization of nerve cells, deterioration of Purkinje cells, neuronal death, deceleration in movement, ataxia, and tremors. GM2-gangliosidosis is characterized by acute neurodegeneration preceded by activated microglia expansion, macrophage, and astrocyte activation, along with the production of inflammatory mediators. However, the mechanism of disease progression in *Hexa−*/*−Neu3−*/*−* mice, relevant to neuroinflammation is poorly understood.

**Method:**

In this study, we investigated the onset and progression of neuroinflammatory changes in the cortex, cerebellum, and retina of *Hexa−*/*−Neu3−*/*−* mice and control littermates by using a combination of molecular genetics and immunochemical procedures.

**Results:**

We found elevated levels of pro-inflammatory cytokine and chemokine transcripts, such as Ccl2, Ccl3, Ccl4, and Cxcl10 and also extensive microglial and astrocyte activation and proliferation, accompanied by peripheral blood mononuclear cell infiltration in the vicinity of neurons and oligodendrocytes. Behavioral tests demonstrated a high level of anxiety, and age-dependent loss in both spatial learning and fear memory in *Hexa−*/*−Neu3−*/*−* mice compared with that in the controls.

**Conclusion:**

Altogether, our data suggest that *Hexa−*/*−Neu3−*/*−* mice display a phenotype similar to Tay-Sachs patients suffering from chronic neuroinflammation triggered by GM2 accumulation. Furthermore, our work contributes to better understanding of the neuropathology in a mouse model of early onset Tay-Sachs disease.

## Background

Tay-Sachs disease (TSD) is a fatal inherited lysosomal storage disorder, principally affecting the brain, which leads to neurological dysfunction. TSD is caused by mutations in the *Hexa* gene, which encodes the *α* subunit of lysosomal β-hexosaminidase α (HEXA), an enzyme that converts GM2 to GM3 ganglioside [1, 2]. Infants with TSD appear healthy at birth, but progressive GM2 accumulation causes loss of motor function and cognition, developmental regression, dystonia, blindness, seizures, and death in childhood [1].

Unlike in TSD patients, the phenotype of *Hexa−/−* knockout (KO) mice was nearly normal, with limited ganglioside storage in the nervous system [1–3]. This result was due to a metabolic bypass in *Hexa−/−* mice, specifically in the NEU3 sialidase-mediated hydrolysis of sialic acid from stored GM2, yielding GA2 ganglioside, which is further degraded by the functional HEXB [3, 4].

Early onset Tay-Sachs disease mouse model (*Hexa−/−* and *Neu3−/−*) mice was generated by crossing of *Hexa−/−* and *Neu3−/−* mice to investigate the role of NEU3 sialidase in GM2 ganglioside degradation [5]. These mice were healthy at birth, but died at 1.5 to 4.5 months of age, showing a similar progression of a very short lifespan like Tay-Sachs patients. It has been shown that abnormal accumulation of GM2 ganglioside in neurons leads to cytoplasmic vacuolation and progressive neurodegeneration, resulting in neuronal death, Purkinje cell depletion, and astrogliosis. *Hexa−*/*−Neu3−*/*−* mice also exhibited neurobehavioral abnormalities, such as growth delay, abnormalities in the skeletal bones, slow movement, ataxia, and tremors. Consequently, the *Hexa−*/*−Neu3*−/*−* mouse model mimics the pathological, biochemical, and clinical abnormalities of the Tay-Sachs patients, and is particularly useful to further understand the pathogenesis, and cellular and molecular mechanisms underlying the progression of TSD [5].

A previous study showed that storage of GM1 and GM2 gangliosides in the CNS led to microgliosis and astrogliosis, and that the degree of inflammation is correlated with increased levels of ganglioside accumulation. While inflammation markers including inflammatory cytokines (TNFα, IL1β, and TGFβ1) were absent in *Hexa*−*/*− mice, they were significantly expressed in the *Hexb*−/− mouse model of Sandhoff disease, GM1 gangliosidosis mouse model [6], and GM2 gangliosidosis patients [7]. Activation of microglia and astrocytes resulted in the production of inflammatory mediators [8]. For instance, the levels of TNF-α pro-inflammatory cytokine were significantly increased in the cerebrospinal fluid of TSD patients [9]. In addition, five inflammation biomarkers, ENA-78, MCP-1, MIP-1α, MIP-1β, and TNFR2 were also detected in the cerebrospinal fluid of patients with infantile and juvenile gangliosidosis [10]. Various lysosomal storage disorders including Niemann–Pick type C disease [11], Gaucher disease [12], mucopolysaccharidosis type I, IIIA, and III [13], and neuronal ceroid-lipofuscinoses [14] also exhibit neuropathological alterations such as prominent microglial and astrocyte activation. A previous study showed that *Hexa*−/−*Neu3*−/− mice exhibited an inflammatory response, with astrogliosis, in the hippocampus, cortex, and cerebellum [5]. In this work, we aimed to further investigate the neuroinflammatory response to GM2 accumulation in the brain and retina of *Hexa*−/−*Neu3*−/− mice, and its effect on behavior during the progression of disease**.**

## Methods

### Animals

*Hexa*−/− on a 129/Sv background [3] and *Neu3*−/− on a C57BL/6 background [15] were bred in the laboratory animal facility, at the Izmir Institute of Technology to generate mice of the target genotype: *WT*, *Hexa*−/−, *Neu3*−/−, and *Hexa*−/−*Neu3*−/−. Breeding and genotyping of these mice was performed as previously described [5]. Genotypes were determined from tail samples using standard PCR procedures and the following primers: *Hexa* primers; *Hexa*F (5″-GGCCAGATACAATCATACAG-3″), *Hexa*R (5″-CTGTCCACATACTCTCCCCACAT-3″) and PGKR (5″-CACCAAAGAAGGGAGCCGGT-3″) and *Neu3* primers *Neu3*F (5″-AAGCAGAGAACATTCTTGAGAGAGCACAGC-3″), *Neu3*R (5″-TCGTGCTTTACGGTATCGCCGCTCCCGATT-3″), and NeoR (5″-GTGAGTTCAAGAGCCATG TTGCTGATGGTG-3″). The mice were maintained at a constant temperature with an alternating 12-h light/dark cycle. Food and water were available ad libitum. The protocols used to perform the experiments described in this work were approved by the Institutional Animal Care and Use Committee of the Izmir Institute of Technology.

### RNA array for cytokine- and chemokine-encoding genes

For a broad analysis of inflammatory gene expression in the cortex and cerebellum of *Hexa*−/− and *Hexa*−/−*Neu3*−/− mice, the mRNA profile of cellular mediators of inflammation, specifically cytokine and chemokines, was assessed using RT^2^ Profiler PCR Array (Qiagen, The Netherlands), according to manufacturer’s instructions. Total RNA was transcribed using the RT^2^ First Strand Kit (Qiagen, The Netherlands). Real-time SYBR green PCR master mix (Qiagen, The Netherlands) was used and PCR cycles were programmed in accordance with the manual. Briefly, (10 min at 95 °C, followed by 40 cycles of 15 s at 95 °C for and 1 min at 60 °C) followed by routine melting curve analysis. All data were normalized to an average of five housekeeping genes, i.e., Gusb (beta-glucuronidase), Hprt (hypoxanthine-guanine phosphoribosyltransferase), Hsp90ab1 (heat shock protein 90 alpha family class B member 1), GADPH (D-glyceraldehyde-3-phosphate dehydrogenase), and Actb (beta-actin). Each sample had a single peak in each reaction at a temperature greater than 80 °C which showed that there is no non-specific product. The threshold cycle changes (△Ct) denote the difference in Ct for the gene of interest based on the Ct level of housekeeping genes. The expression of each gene was normalized using the mean expression of five different housekeeping genes in the array. Fold changes in the expression level of genes were obtained according to the 2^−ΔΔCT^ method. Genes that had positive or negative fold changes of more than 1.5-fold with a *p* value smaller than 0.05, were considered to be statistically significant.

### Immunofluorescence analysis

Mice were deeply anesthetized (ketamine/xylazine; 200/10 mg/kg) and perfused with fixative (4% paraformaldehyde in PBS, pH 7.4). Brains were removed and post-fixed in the same fixative solution, overnight at 4 °C, and then sequentially treated with 10%, 20%, and 30% sucrose in PBS. Brains were embedded in Tissue-Tek OCT compound (Sakura Finetechnical, Tokyo, Japan), and frozen at −80 °C. Frozen brain specimens were sectioned at 10 μm thickness, and mounted on a HistoBond®microscope slides (Marienfeld, Germany) at −20 °C, using a Leica cryostat. Coronal brain slices (10 μm) obtained from the mice at the indicated ages were treated with ice-cold acetone, and then, blocked using blocking buffer (4% BSA, 10% goat serum, 0.3% Triton X-100, and 0.3 M glycine in PBS), for 1 h at room temperature in a humidified chamber. Anti-CD45 (1:300; Santa Cruz Biotechnology, USA), anti-Moma2 (1:50; Abcam, USA), anti-LAMP1 (1:500; Abcam, USA), anti-CNPase (1:50; Cell Signaling Technology, The Netherlands), and anti-NeuN (1:100; Cell Signaling Technology, The Netherlands) were diluted in blocking buffer and applied overnight at 4 °C. The following Alexa Fluor conjugated secondary antibodies was used to visualize target primary antibodies; goat anti-rabbit Alexa Fluor 568 (Abcam, USA), goat anti-rabbit Alexa Fluor 488 (Abcam, USA), goat anti-rat Alexa Fluor 568 (Abcam, USA). Anti-IL6 (1:250; Santa Cruz Biotechnology, USA) antibody was diluted in the blocking buffer (4% BSA, 10% goat serum, 0.3% Triton X-100, and 0.3 M glycine in PBS) overnight and it was labeled with donkey anti-goat Alexa Fluor 555 (Abcam, USA). The slides were mounted with Fluoroshield mounting medium with DAPI (Abcam, USA) and images were obtained using a light microscope (Bx53, Olympus Corporation, Germany) equipped with a manually controlled specimen, a color camera (DP73, Olympus Corporation, Germany), a fluorescent light source (U-RFL-T, Olympus Corporation, Germany), and image analysis software (cellSens Entry, Olympus Corporation, Germany).

### Retina analysis

Eyes were removed and fixed for 24 h with 4% PFA, at room temperature. Retinas were prepared as flattened whole mounts, by making four radial cuts, and labeled with anti-lectin (20 μg/mL) (Vector Laboratories, USA) and anti-phalloidin (1:100) (Biotium, USA) antibodies. Retinas were permeabilized by washing in PBST for 30 min, then, washed them in PBS 3 times for 10 min and, finally, anti-lectin was applied at 4 °C overnight. The following day, they were washed in PBS 3 times for 5 min and incubated in anti-phalloidin at 4 °C overnight. Retinas were mounted with an anti-fade reagent containing Hoechst-33258 to label nuclei (Molecular Probes, USA). Twenty micrometer-thick sagittal sections were assessed for potential anatomical alterations. Finally, whole-mount retinas and sagittal sections were imaged using a light microscope (× 400, Eclipse E600, Nikon Instruments Inc., Japan) equipped with a manually controlled specimen stage for X, Y, and Z axis, a color camera (model DXM1200, Nikon Instruments Inc., Japan), a fluorescent light source (HB-10104AF, Nikon Instruments Inc., Japan), and the image analysis software (NIS-Elements, Version 3.22, Nikon Instruments Inc., Japan).

### Passive avoidance test

This test was conducted as previously described [16]. Briefly, the apparatus, which is divided has both into a lit compartment and a dark compartment, with a vertical sliding door between the two. Animals were allowed to explore both compartments on the first day. Each mouse was placed into the bright compartment, and after 30 s the central door opened allowing it to migrate into the dark compartment, an environment which they generally prefer. The following day, upon entry into the dark compartment, the door was closed and the mouse received a mild electric shock (0.2 mA for 2 s). On the final day, animals were given the same choice to enter the dark compartment (300 s maximum time). Latency to cross through the door between compartments was scored with ShutAvoid v1.8 (Harvard Apparatus, USA).

### Morris water maze test

Spatial learning and memory were assessed as previously described [16]. The water maze consists of a large circular pool (diameter 140 cm; depth, 45 cm) filled with an opaque solution of dry milk in water (22 °C), which is located in a room with numerous visual cues. Spatial acquisition was organized in 3 training sessions (days 1-3) with a visible platform (1 cm above the platform). Each mouse has 60 s to reach the platform. The location of cues and platform were changed every day, and the mice were released into the water at 1 of 3 different locations. On days 4-8, animals received reversal training with an invisible platform (1 cm below the water surface). For this training, the platform location was kept constant, and mice were placed into the water at 1 of 3 different locations and given 90 s to find the platform. Measurements were acquired with a Sony camera (model SSC-G18), centrally positioned above the water tank. Behavioral differences were analyzed using the Panlab SMART Video Tracking System v0.3 (Harvard Apparatus, USA). Animals were allowed to dry under a heat lamp after each trial to avoid hypothermia, and all experiments were started at the same time each day.

### Grip strength

Mice were subjected to a forelimb grip strength test, with a Grip Strength Meter (IITC Life Science, USA). The gage was reset to 0 g after stabilization, and each mouse was encouraged to grab a T-shaped bar. The connection of the bar to a digital force transducer allowed quantification of strength as a pull force in grams. The order of mice tested was randomized, and the experimenter was blinded to the genotypes of mice.

### Open field test

The apparatus for this assay consisted of a 40 × 40 cm surface area and was surrounded from all sides by a 40 cm transparent wall. A digital camera was mounted directly above the apparatus. Mice were placed in one of the corners of the open field and allowed to explore undisturbed for 5 min. Behavioral differences were analyzed using the Panlab SMART Video Tracking System v0.3 (Harvard Apparatus, USA).

### Statistical analysis

The GraphPad QuickCalcs (GraphPad Software, USA) software was used for statistical analysis. All values are expressed as the mean ± S.E.M. Differences were tested using one-way ANOVA for behavioral analysis and immunofluorescence analysis. qPCR data were analyzed by unpaired *t* test. A *p* value of less than 0.05 was considered to represent statistical significance.

## Results

### Altered levels of the inflammatory cytokines and chemokines

Neuroinflammation involves the release of both inflammatory cytokines and chemokines [17]. Therefore, we first evaluated the transcript levels of pro- and anti-inflammatory cytokines and chemokines in the cortex and cerebellum of 4.5-month-old *Hexa*−/− and *Hexa*−/−*Neu3*−/− mice. We used a preformatted gene pathway array to compare the expression of 84 inflammatory chemokine, cytokine, and interleukin receptor genes relative to that in *Hexa*−/− mice. After disregarding unregulated, non-detectable gene products and evaluating the relative levels of gene expression across all samples, we identified 42 inflammatory genes, which we categorized into 3 distinct groups.

**Fig. 1:**
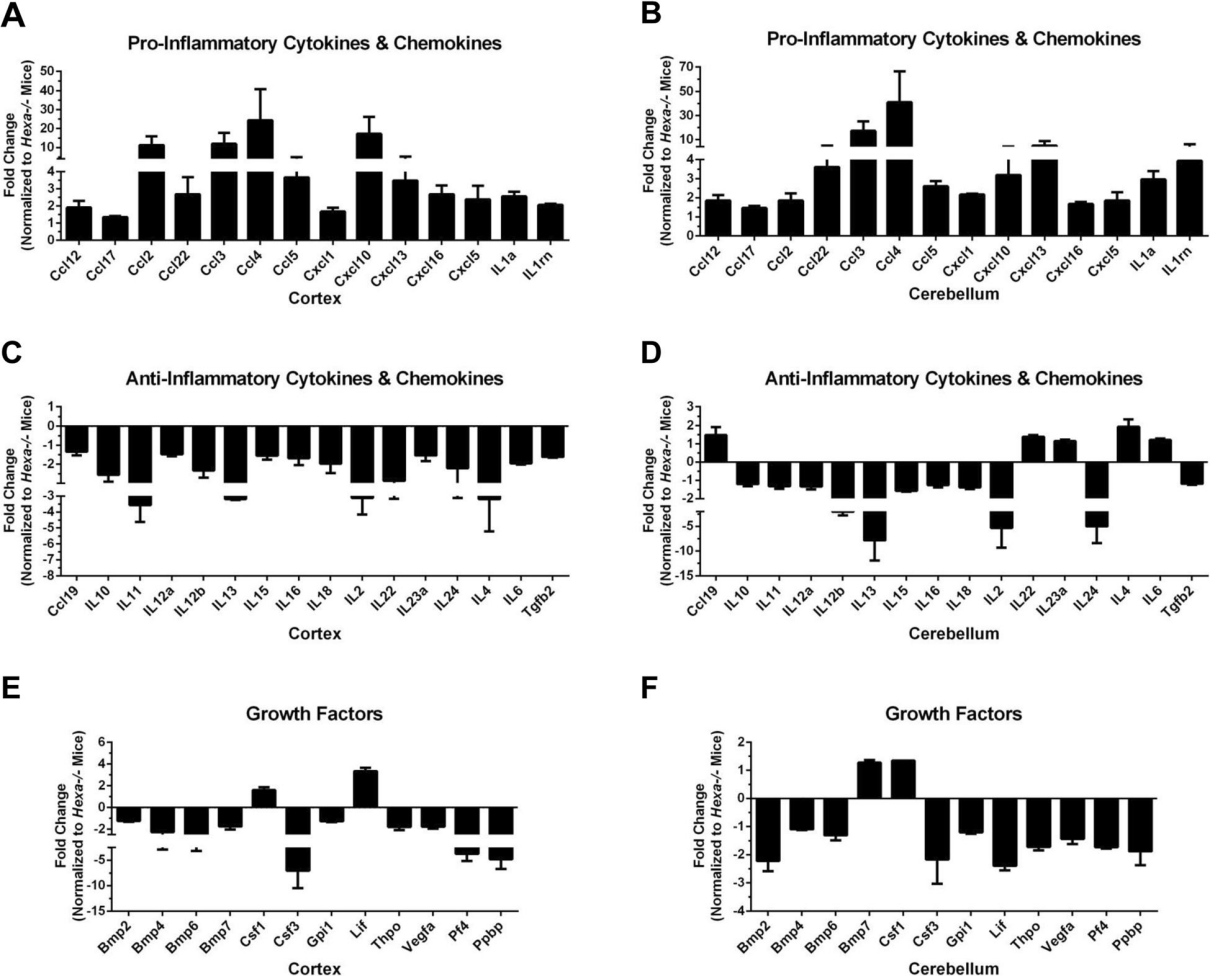
Relative expression levels of inflammatory cytokines and chemokines (**a** and **b**), anti-inflammatory cytokines and chemokines (**c** and **d**), and growth factors (**e** and **f**) in the cortex and cerebellum of 4.5-month-old *Hexa*−/−*Neu3*−/− mice (*n* = 2) normalized to *Hexa*−*/*− mice (*n* = 2)

Pro-inflammatory cytokines and chemokines, secreted mostly by activated macrophages in microglial cells, are involved in the upregulation of inflammatory reactions [18]. Our data showed that *Hexa*−/−*Neu3*−/− mice displayed an altered expression profile for the pro- and anti-inflammatory cytokines, as well as chemokines in both of the cortical (Fig. 1a) and cerebellar (Fig. 1b) regions. Very large increases were seen in Ccl2 (11.3-fold), Ccl3 (12-fold), Ccl4 (24.4-fold), and Cxcl10 (17.2-fold) in the cortex of *Hexa*−*/*−*Neu3*−*/*− mice (Fig. 1a). Ccl3 (17.2-fold), Ccl4 (41-fold), and Cxcl13 (fivefold) were predominantly expressed in the cerebellum. It has been established that Ccl2 cytokine displays chemotactic activity for monocytes, lymphocytes, and neutrophils, while Ccl3 is involved in the migration of monocytes, lymphocytes, and neutrophils together with Ccl2 [18] and activates T cells and macrophages with Ccl4 [19]. Increased expression of Ccl3 and Ccl4 in the brain and cerebellum were also observed in different lysosomal storage diseases such as MPSIIIA [20] and MPSIIIB [21], and Gaucher disease [22, 23]. In addition, Cxcl10, an inflammatory chemokine produced by astrocytes, which recruits activated T lymphocytes by increasing their migration to the site of tissue damage, is increased in both cortex and cerebellum of *Hexa*−/−*Neu3*−/− mice [24, 25].

Anti-inflammatory cytokines and chemokines regulate the expression of pro-inflammatory cytokines to arrest or slow down the immune response [26]. We found not only an upregulation of pro-inflammatory cytokines and chemokines in *Hexa*−/−*Neu3*−/− mice (Fig. 1a, b) but also a downregulation of anti-inflammatory cytokines and chemokines, which provides a more complete picture of the inflammatory conditions in the cortex (Fig. 1c) and cerebellum (Fig. 1d). Analysis of anti-inflammatory cytokines and chemokines revealed a significant decrease in the transcript levels of IL10 (2.6-fold), IL11 (3.6-fold), IL13 (3.2-fold), IL2 (3.1-fold), IL22 (2.9-fold), and IL4 (3.2-fold) in the cortex of *Hexa*−/−*Neu3*−/− mice compared to *Hexa*−/−. In addition, we found that IL10 (1.2-fold), IL11 (1.3-fold), IL13 (7.8-fold), IL2 (5.3-fold), and IL24 (fivefold) were significantly decreased in the cerebellum of *Hexa*−/−*Neu3*−/− mice, compared to *Hexa*−/− mice. IL10 protects tissues by preventing excessive inflammation [27], and IL13 has a similar immunosuppressant activity [28]. Decreased expression of IL10 and IL13 in the cortex and cerebellum was thought to contribute to the neuroinflammation seen in *Hexa*−/−*Neu3*−/− mice (Fig. 1c, d).

**Fig. 2:**
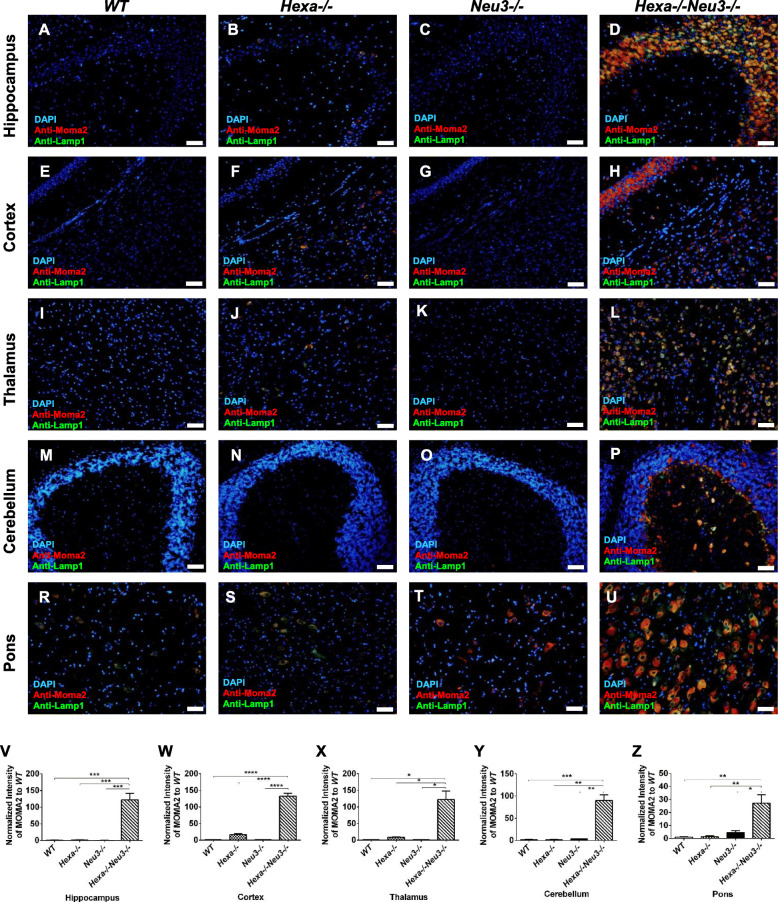
Immunohistochemical analysis to detect microglial activation. The sections from the hippocampus (**a**, **b**, **c**, and **d**, respectively), cortex (**e**, **f**, **g**, and **h**, respectively), thalamus (**i**, **j**, **k**, and **l**, respectively), cerebellum (**m**, **n**, **o,** and **p**, respectively), and pons (**r**, **s**, **t**, and **u**, respectively) of 4.5-month-old *WT*, *Hexa*−/−, *Neu3*−/−, and *Hexa*−/−*Neu3*−/− mice were stained with anti-Moma2 antibody (red), anti-lamp1 (green), and DAPI (blue). A yellow signal signifies the colocalization of Moma2 and lamp1 as an activated microglial cell. The histograms represent the quantification of activated microglial cells in the hippocampus (**v**), cortex (**w**) thalamus (**x**), cerebellum (**y**), and pons (**z**). Scale bar = 50 μm. The data are represented as the mean ± SEM. One-way ANOVA was used for statistical analysis (*p < 0.05, **p < 0.025, ***p < 0.01, and ****p < 0.001)

Expression levels of growth factors in the cortex (Fig. 1e) and cerebellum (Fig. 1f) of *Hexa*−/−*Neu3*−/− mice were also analyzed. We found that the expression ratio of Bmp2 (1.2-fold), Bmp4 (2.3-fold), Bmp6 (2.5-fold), Bmp7 (1.7-fold), and Csf3 (7.1-fold) in the cortex was lower than *Hexa*−/− mice, whereas the expression ratio of Csf1 (1.6-fold) and Lif (3.4-fold) was higher. In the cerebellum, Bmp2 (2.2-fold), Bmp4 (1.1-fold), Bmp6 (1.3-fold), and Csf3 (2.2-fold) were decreased, while Csf1 (1.3 fold) was increased compared with that in *Hexa*-/- mice. Csf1, a cytokine that controls the production, differentiation, and function of macrophages [29], was increased in the cortex (1.6-fold) and cerebellum (1.3-fold) of *Hexa*−/−*Neu3*−/− mice, which may account for the higher number of macrophages seen in these regions (Fig. 2h, p).

### Microglial activation

Microglial cells are the immune cells of the CNS responsible for sensing stress signals released by damaged or dying neurons [19]. They migrate to sites of injury where they release cytokines to promote the removal of dead and dying cells by phagocytosis [30]. Once stimulated, they can act to activate neighboring microglia, astrocytes, neurons, or oligodendrocytes. Several studies have shown that the accumulation of undegraded macromolecules in lysosomal storage disorders, including gangliosidosis, results in activation of a neuroinflammatory response in the CNS [17, 19]. To visualize the location of active microglia, brain sections from *WT*, *Hexa*−/−, *Neu3*−/−, and *Hexa*−/−*Neu3*−/− mice were immunostained with anti-Moma2 and anti-Iba1 antibodies, which are specific to the activated microglial/macrophage system [31–33]. The number of Moma2-positive cells was significantly increased in the hippocampus, cortex, thalamus, and pons of 4.5-month-old *Hexa*−/−*Neu3*−/− compared with that in *WT*, *Hexa*−/−, and *Neu3*−/− mice (Fig. 2). We found significantly increased microgliosis in the brains of 4.5-month-old *Hexa*−/−*Neu3*−/− mice compared with that in age-matched *Hexa*−/− mice, with the following ratios: approximately 60-fold in hippocampus (Fig. 2v), ninefold in the cortex (Fig. 2w), 13-fold in thalamus (Fig. 2x), and 19-fold in pons (Fig. 2z). In the cerebellum, microgliosis was observed only in *Hexa*−/−*Neu3*−/− mice (Fig. 2p, y). Significantly increased microgliosis was also observed in 2.5-month-old *Hexa*−*/*− *Neu3*−*/*− mice compared with that in age-matched *Hexa*−*/*− mice, with the following ratios: approximately 37-fold in the hippocampus (Supp. Fig. 1V), fourfold in the cortex (Supp. Fig. 1W) and fivefold in the thalamus (Supp. Fig. 1X). Only *Hexa*−*/*−*Neu3*−*/*− mice displayed microgliosis in the cerebellum (Supp. Fig. 1P, Y). There was no microglial activation in the hippocampus, cortex, thalamus, and cerebellum of *WT* and *Neu3*−*/*− mice, with the exception of in the pons area. Iba1 immunostaining also showed a significant expansion of microglia in 4.5-month-old *Hexa*−*/*−*Neu3*−*/*− mice compared to age-matched WT mice with the following ratios: 4.5-fold in the hippocampus (Supp. Fig. 2D), 82-fold in the cortex (Supp. Fig. 2G), and sixfold in the pons (Supp. Fig. 2P). Virtually, Moma2+/Iba1+ cells displayed amoeboid morphology with increased cell area in the *Hexa*−*/*−*Neu3*−*/*− mice brain, which is a characteristic feature of maximally activated microglia [34]. The phagocytic Moma2+ microglia were detected as 52%, 25%, 39%, 44%, and 42% of Moma2+ cells in the hippocampus, cortex, thalamus, cerebellum, and pons, respectively (Fig. 2z). The Moma2+/Iba1− signals might indicate permeabilized monocyte and macrophages into the CNS. All of these results show that *Hexa*−*/*−*Neu3*−*/*− mice brain includes an activated microglial/macrophage system in the CNS. The presence of neuroinflammatory conditions such as astrogliosis in the hippocampus, cortex, and cerebellum of 4.5-month-old *Hexa*−*/*−*Neu3*−*/*− mice has been previously reported [5]. In this study, we showed that activated astrocytes are present in the hippocampus, cortex, and cerebellum of not only 4.5-month-old but also 2.5-month-old *Hexa*−*/*− *Neu3*−*/*− mice (Supp Fig. 3).

### Increased infiltration of peripheral blood mononuclear cells (PBMCs) in the brain

Neuroinflammation contributes to disease progression in several brain disorders. Specifically, activated immune cells induce the infiltration of T and B cells into the CNS, promoting the release of chemokines and stimulating the migration of more immune cells to the CNS via a disrupted blood-brain-barrier (BBB) [35]. Previous studies showed that neuroinflammation leads to significant infiltration of PBMC in the brain of the Sandhoff mouse model [36].

**Fig. 3:**
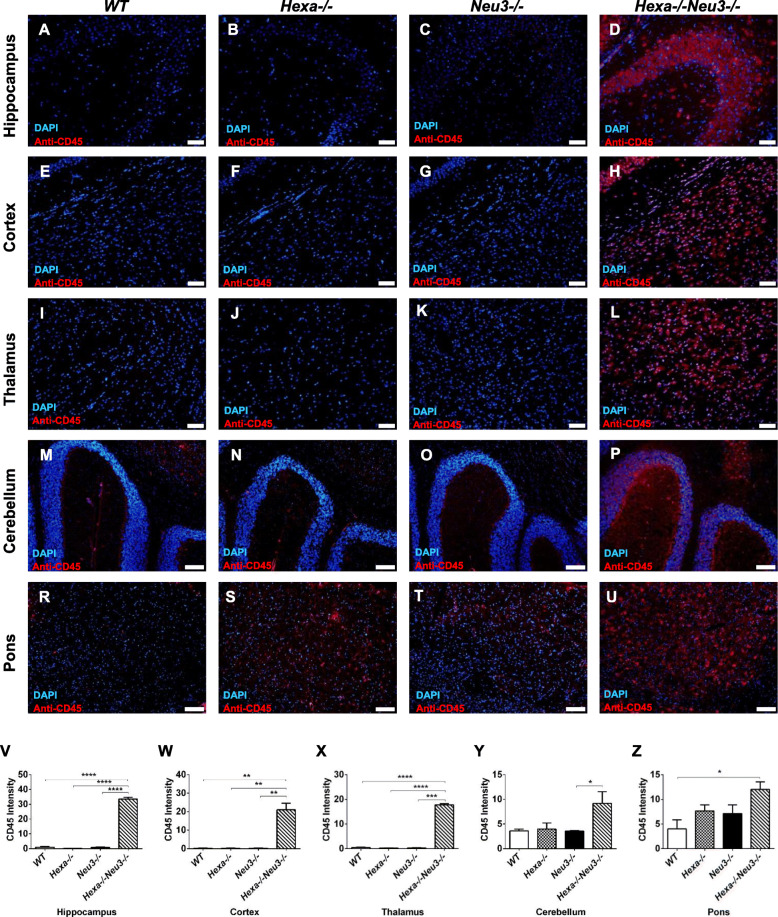
Immunohistochemical analysis of activated PBMC. The sections from the hippocampus (**a**, **b**, **c**, and **d**, respectively), cortex (**e**, **f**, **g**, and **h**, respectively), thalamus (**i**, **j**, **k**, and **l**, respectively), cerebellum (**m**, **n**, **o,** and **p**, respectively), and pons (**r**, **s**, **t**, and **u**, respectively) of 4.5-month-old *WT*, *Hexa*−/−, *Neu3*−/−, and *Hexa*−/−*Neu3*−/− mice were stained with anti-CD45 antibody (red) and DAPI (blue). The histograms represent the quantification of CD45(+) cell number in the hippocampus (**v**), cortex (**w**), thalamus (**x**), cerebellum (**y**), and pons (**z**). Scale bar = 50 μm for the hippocampus, cortex, and thalamus; 100 μm for cerebellum and pons. The data are represented as the mean ± SEM. One-way ANOVA was used for statistical analysis (*p < 0.05, **p < 0.025, ***p < 0.01, and ****p < 0.001)

To analyze the presence of activated PBMC due to significantly increased Ccl2, Ccl3, and Cxcl10 in both the cortex and cerebellum of *Hexa*−*/*−*Neu3*−*/*− mice, 10 μm coronal brain sections from 4.5-month-old *WT*, *Hexa*−/−, *Neu3*−/−, and *Hexa*−/−*Neu3*−/− were immunostained with anti-CD45 antibody and anti-Iba1. The average number of CD45-positive cells including microglial cells in the hippocampus, cortex, and thalamus of *Hexa*−/−*Neu3*−/− mice was significantly increased compared with that in *WT*, *Hexa*−/− and *Neu3*−/− (Fig. 3). Similarly, activated PBMCs were significantly increased in the cerebellum (2.5-fold) of *Hexa*−/−*Neu3*−/− mice (Fig. 3p) compared with that in *Neu3*-/- mice (Fig. 3y) as well as in the pons (threefold) of *Hexa*−/−*Neu3*−/− mice (Fig. 3u) compared with that in *WT* mice (Fig. 3z). Readily detectable numbers of CD45-positive cells were also identified in the cerebellum and pons of *WT* (Fig. 3m, r, respectively) *Hexa*−/− (Fig. 3n, s, respectively) and *Neu3*−/− (Fig. 3o, t, respectively). CD45 is known to be upregulated in activated microglial cells. Immunohistochemical analysis for CD45 expression and its colocalization with Iba-1 revealed a widespread distribution of positive cells in Hexa−/−Neu3−/− mice brain with 83%, 68%, 75%, 48%, and 54% colocalization level in the hippocampus, cortex, thalamus, cerebellum, and pons, respectively. CD45+/Iba1+ cells might represent microglia and CD45+/Iba1− cells might indicate PBMC+ cells in *Hexa*−/−*Neu3*−/− brain (Supp. Fig. 4). Thus, the progression of TSD could be associated with disruption of the blood-brain-barrier, and widespread infiltration of inflammatory immune cells into the CNS.

### Decreased number of oligodendrocyte and neurons

**Fig. 4:**
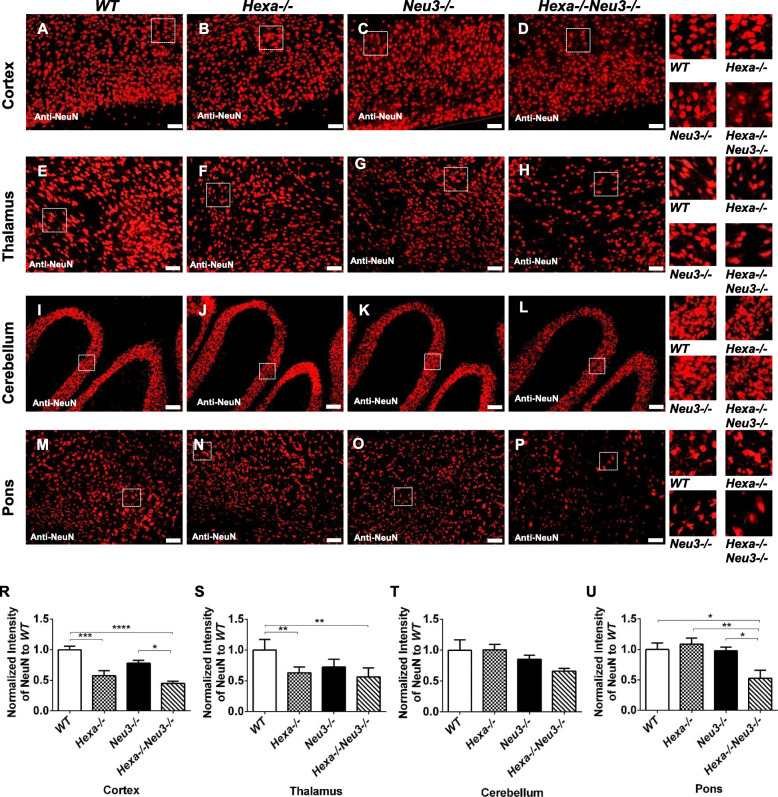
Neuronal density detection in cortex (**a**, **b**, **c**, and **d**, respectively), thalamus (**e**, **f**, **g**, and **h**, respectively), cerebellum (**i**, **j**, **k**, and **l**, respectively), and pons (**m**, **n**, **o**, and **p**, respectively) of 4.5-month-old *WT*, *Hexa*−/−, *Neu3*−/−, and *Hexa*−/−*Neu3*−/− mice. They were stained with anti-NeuN antibody (red) and DAPI (blue). The histograms represent quantification of neuronal density in the cortex (R), thalamus (S), cerebellum (T), pons (U). Scale bar = 50 μm for cortex and thalamus; 100 μm for cerebellum and pons. The data are represented as the mean ± SEM. One-way ANOVA was used for statistical analysis (*p < 0.05, **p < 0.025, ***p < 0.01, and ****p < 0.001)

To characterize the degree to which neuroinflammation affected neuronal cells and oligodendrocytes, 10 μm coronal brain sections from 2.5 and 4.5-month-old *WT*, *Hexa*−/−, *Neu3*−/−, and *Hexa*−/−*Neu3*−/− mice were immunostained with NeuN and CNPase antibodies, which recognize nonspecific neurons and oligodendrocytes, respectively. As shown in Fig. 4, 4.5-month-old *Hexa*−*/*−*Neu3*−*/*− mice showed a loss in neuronal density in different regions of CNS compared to age-matched control groups. The analysis demonstrated that the number of NeuN-positive neurons was significantly reduced by approximately 50% in the cortex, 40% in the thalamus, and 50% in the pons of *Hexa*−/−*Neu3*−/− mice (Fig. 4d, h, p, respectively) compared with that in *WT* mice (Fig. 4a, e, m, respectively). There was no significant change in neuronal density, in the granular layer of the cerebellum (Fig. 4t). Furthermore, there was no significant change in the number of neurons in 2.5-month-old *Hexa*−/−*Neu3*−/− compared with that in age-matched control groups in different regions of the CNS (Supp. Fig. 5). Loss of NeuN immunoreactivity could be explained by increased neuronal death in damaged areas of the brain [37]. Thus, a decrease in NeuN immunostaining correlates with increased numbers of apoptotic cells. In a previous study, a TUNEL assay showed that there was apoptotic cell death in the brain of *Hexa*−/−*Neu3*−/− mice compared with that in *WT*, *Hexa*−/−, and *Neu3*−/− mice [5]. Furthermore, NeuN-positive neurons in the cortex (Fig. 4d), thalamus (Fig. 4h), and hippocampus (Fig. 4p) appeared swollen, with accumulated storage material as previously shown in the Sandhoff mice brain [38].

**Fig. 5:**
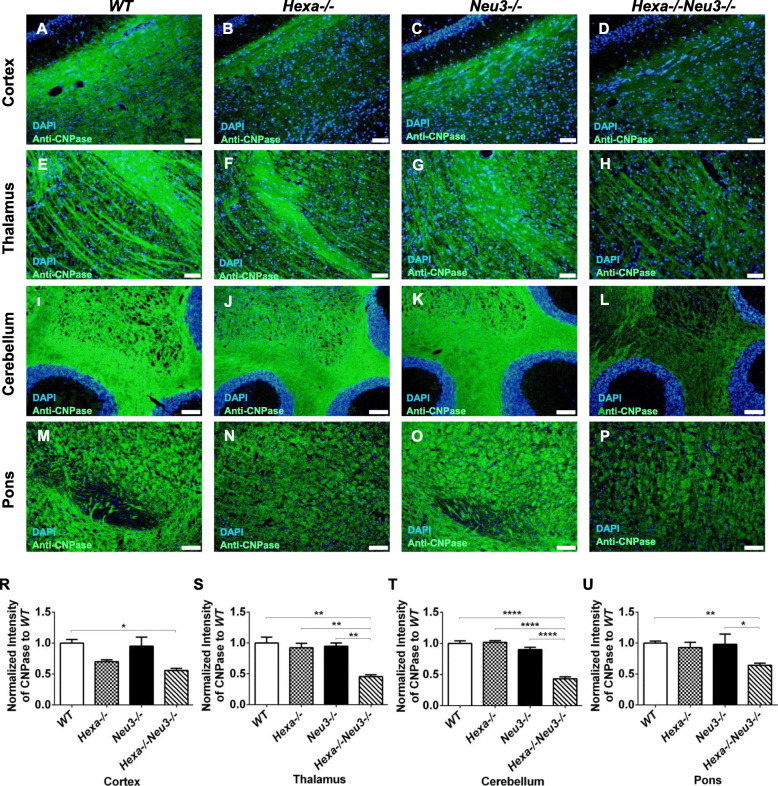
Immunohistochemical staining for oligodendrocytes. The sections from the cortex (**a**, **b**, **c**, and **d**, respectively), thalamus (**e**, **f**, **g**, and **h**, respectively), cerebellum (**i**, **j**, **k**, and **l**, respectively), and pons (**m**, **n**, **o**, and **p**, respectively) of 4.5-month-old *WT*, *Hexa*−/−, *Neu3*−/−, and *Hexa*−/−*Neu3*−/− mice were stained with anti-CNPase (red) and DAPI (blue). The histograms represent the quantification of oligodendrocytes in the cortex (R), thalamus (S), cerebellum (T), pons (U). Scale bar = 50 μm for cortex and thalamus; 100 μm for cerebellum and pons. The data are represented as the mean ± SEM. One-way ANOVA was used for statistical analysis (*p < 0.05, **p < 0.025, and ****p < 0.001)

Oligodendrocytes are types of glial cells that function in the formation of myelin, which then supplies support and insulation to axons [30]. Oligodendrocytes provide axons with myelin sheaths, and have the ability to renew their myelin sheaths three times within a day. Myelination is essential for optimal signal transduction in the CNS. Activated microglia produces various pro-inflammatory mediators, chemokines, and cytokines. In such environments, oligodendrocytes are particularly susceptible to microglia-derived factors, resulting in the production of poor-quality myelin sheaths and oligodendrocyte death [39]. As shown in Fig. 5, Hexa−/−Neu3−/− mice showed lower numbers of 2′,3′-cyclic-nucleotide 3′-phosphodiesterase-positive cells compared with age-matched WT, *Hexa*−/−, and *Neu3*−/− mice. The number of oligodendrocytes was reduced by approximately 45% in the cortex, 55% in the thalamus and cerebellum, and 35% in the pons area of *Hexa*−/−*Neu3*−/− mice (Fig. 5d, h, l, p, respectively) compared with that in *WT* mice (Fig. 5a, e, i, m, respectively). The number of oligodendrocytes was significantly decreased in the cortex of *Hexa*−/− mice, by approximately 30% compared with that in *WT* mice. We observed no significant changes in the number of oligodendrocytes in the thalamus, cerebellum, and pons of *Hexa*−/− (Fig. 5f, j, n, respectively) and *Neu3*−/− (Fig. 5g, k, o, respectively) mice, compared with that in *WT* mice. These results are consistent with previous studies. Gene expression profile studies in the cerebral cortex of normal and GM2 gangliosidosis (Tay-Sachs and Sandhoff) patients revealed that the myelin basic protein gene, expressed by oligodendrocytes, was also significantly depressed [8]. Sialiated gangliosides, especially GD1a and GT1b, are present on the axonal membrane and interact with the myelin-associated glycoprotein (MAG) on the periaxonal surface to promote myelin sheath stability [40]. GM2 is also a sialic acid-containing ganglioside; thus, the accumulation of GM2 could lead to instability of myelin sheath. We found no significant changes in the number of oligodendrocytes in 2.5-month-old *Hexa*−/−*Neu3*−/− mice compared with that in age-matched control groups (Supp. Fig. 6).

### Increased number of microglia in the retina

**Fig. 6:**
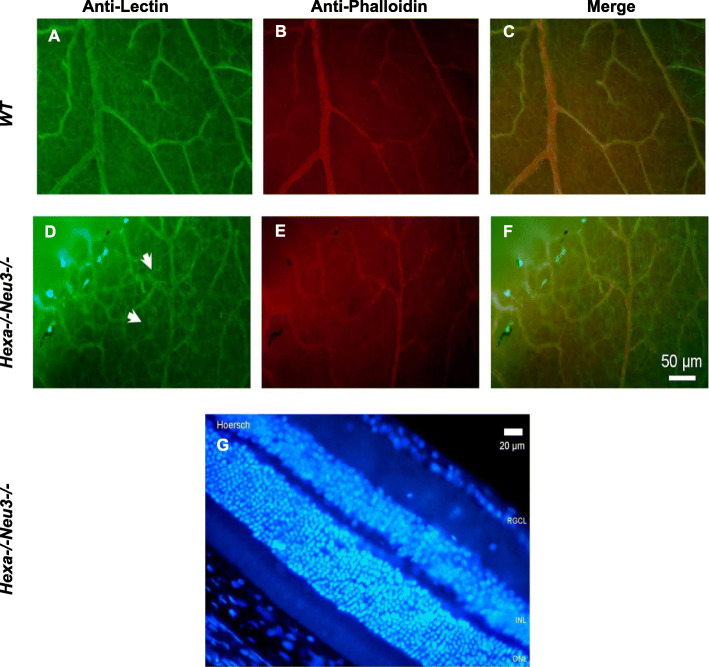
Immunohistochemical analysis to detect glial activation in the retina. The retinas of 4.5-month-old *WT* (**a**, **b**, and **c**) and *Hexa*−/−*Neu3*−/− (**d**, **e**, and **f**) mice were stained with anti-lectin (green) and anti-phalloidin (red) antibody. The white arrow indicates the glial cells. The sagittal sections of *Hexa*−/−*Neu3*−/− mice retina (G) were stained with Hoechst (blue). Scale bar = 50 μm in **a**, **b**, **c**, **d**, **e**, and **f**; 20 μm in **g**

Neuroinflammation or neurodegeneration leads to reactive gliosis in the retina through hypertrophy, causing thickening and enlargement of processes via Müller cells and astrocytes [41]. To characterize the neuroinflammatory conditions affecting the retina, coronal eye sections from 4.5-month-old *WT*, and *Hexa*−/−*Neu3*−/− mice were immunostained with anti-lectin (vessels and glia) and anti-phalloidin antibodies (vessels and actin filaments). We found significantly higher numbers of microglial cells in the retinas of *Hexa*−/−*Neu3*−/− mice compared with that in *WT* mice (Fig. 6a, d). Microglial staining was prominent around the vessels. However, sagittal sections of *Hexa*−/−*Neu3*−/− mice showed no anatomical alterations in their retinal layers (Fig. 6g).

### Increased IL-6 expression in neurons

Elevated IL6 (pro-inflammatory cytokine) production due to activated microglia has been observed in lysosomal storage disorders, e.g., in the brains of the Gaucher disease mouse model, in the serum of Gaucher, Fabry, and mucopolysaccharidosis type IVA patients [19]. To detect neuronal expression of IL6, brain sections of 4.5-month-old *WT*, *Hexa*−*/*−, *Neu3*−*/*−, and *Hexa*−*/*−*Neu3*−*/*− mice were immunostained with an anti-IL6 antibody. The number of IL6-positive cells was significantly increased in the hippocampus, cortex, thalamus, and pons of *Hexa*−*/*−*Neu3*−*/*− compared with that in *WT*, *Hexa*−*/*−, and *Neu3*−*/*− (Supp. Fig.7). There was no significant change in the number of IL6-positive cells in the cerebellum of *WT*, *Hexa*−*/*−, *Neu3*−*/*−, and *Hexa*−*/*−*Neu3*−*/*− mice. We found an approximately threefold increase in IL6-positive cells in the hippocampus (Supp. Fig. 7V), cortex (Supp. Fig. 7W), and thalamus (Supp. Fig. 7X) of *Hexa*−*/*−*Neu3*−*/*− mice compared with that in age-matched *Hexa*−*/*− mice.

### Impairments in spatial learning and memory

**Fig. 7:**
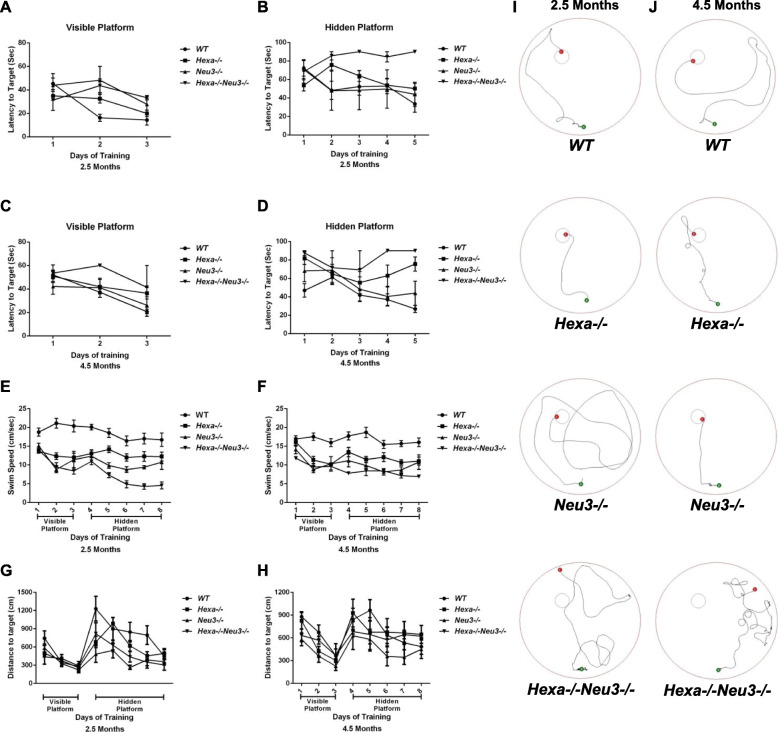
Morris water maze test. Latency to target for the visible platform (**a** and **c**) and hidden platform (**b** and **d**), swim speed (**e** and **f**), and distance to the target (**g** and **h**) were analyzed. Typical swim patterns of 2.5- and 4.5-month-old mice on day 8 were shown (**i** and **j**, respectively). The data are presented as means + SEM. A 2.5-month-old *WT* (*n* = 4), *Hexa*−/− (*n* = 8), *Neu3*−/− (*n* = 3), and *Hexa*−/−*Neu3*−/− (*n* = 3) mice; 4.5-month-old *WT* (*n* = 6), *Hexa*−/− (*n* = 3), *Neu3*−/− (*n* = 3), and *Hexa*−/−*Neu3*−/− (*n* = 3)

Upregulation of cytokines and their receptors within the CNS during inflammation, and concomitant effects on brain function, have been reported [6]. The Morris water maze task was used to detect spatial learning and memory deficits [42–45]. We used a two-way ANOVA to analyze the data. Here, we report that both 2.5- and 4.5-month-old *Hexa*−/−*Neu3*−/− mice displayed deficits in spatial learning and memory. Also, we found that in the first 3 days of training *WT* (*p* < 0.001), *Hexa*−/− (*p* < 0.025), and *Neu3*−/− (*p* < 0.05) mice learned to use the visual clues to quickly reach the visible platform, whereas *Hexa*−/−*Neu3*−/− mice took a longer time to swim toward it (Fig. 7a, c). All groups initially had difficulty in finding the exact location of the hidden platform on day 4. While *WT*, *Hexa*−/−, and *Neu3*−/− mice groups quickly improved their ability to find the platform, both (*p* < 0.01) 2.5- and (*p* < 0.025) 4.5-month-old *Hexa*−/−*Neu3*−/− mice were not able to learn the location of the hidden platform compared to *WT*. Also, 2.5-month-old *WT* (*p* < 0.025), Hexa-/- (*p* < 0.05), and Neu3-/- (*p* = ns) mice and 4.5-month-old *WT* (*p* < 0.025) and *Neu3*−/− (*p* = ns) mice spent continuously less time, from day one to five, to find the hidden platform (Fig. 7b, d). Our data showed that *WT*, *Hexa*−/−, and *Neu3*−/− mice used distal clues more efficiently than *Hexa*−/−*Neu3*−/− mice.

Swimming speeds recorded in the Morris water maze were significantly lower in 2.5- and 4.5-month-old *Hexa*−/−*Neu3*−/− (7.8 ± 0.8; 8.7 ± 0.6, respectively) mice compared with that in age-matched *WT* (18.6 ± 1.3, *p* < 0.0001; 16.8 ± 1, *p* < 0.0001, respectively), Hexa-/- (12.7 ± 0.9, *p* < 0.0001; 12 ± 0.9, *p* < 0.001, respectively), and *Neu3*−/− (10.8 ± 1.2, *p* < 0.05; 20.2 ± 1.3, *p* = ns, respectively) (Fig. 7e, f). However, the average distance spent to find the target platform was similar in *WT*, *Hexa*−/−, *Neu3*−/−, and *Hexa*−/−*Neu3*−/− mice in both 2.5 and 4.5-month-old mice groups (Fig. 7g, h).

Typical swim patterns revealed that both 2.5- and 4.5-month-old *Hexa*−/−*Neu3*−/− mice were unable to find the escape platform by the final day of the test, whereas age-matched *WT*, *Hexa*−/−, and *Neu3*−/− mice were successful at using distal clues to find it. In addition, 4.5-month-old *Hexa*−/−*Neu3*−/− mice showed more anxiety-related behaviors compared with that in WT and single deficient *Hexa*−/− and *Neu3*−/− mice (Fig. 7i, j).

The 5 min open-field test was performed to measure levels of anxiety [46]. A significant difference was detected between *WT* and *Hexa*−/−*Neu3*−/− mice (*p* < 0.001 for 2.5-month; *p* < 0.025 for 4.5-month) in an age-independent manner. Moreover, *Hexa*−/−*Neu3*−/− (291.2 ± 1.8 s for 2.5-month; 288.4 ± 2.1 s for 4.5-month) mice spent most of the time in the periphery of the open field, compared to *WT* (256.3 ± 7.5 s for 2.5 month; 268.3 ± 4.1 s for 4.5 months), *Hexa*−/− (275.4 ± 3.2 s for 2.5 month; 284.4 ± 3.1 s for 4.5 months), and *Neu3*−/− (273 ± 7.2 s for 2.5 month; 277.2 ± 3.3 s for 4.5 months). The walking patterns and the amount of time spent in the center and the periphery of the open field area indicated that both, 2.5- and 4.5-month-old *Hexa*−/−*Neu3*−/− mice displayed more anxiety-related behaviors, compared to the other genotypes (Supp. Fig. 8).

### Deficits in cognitive learning and memory

**Fig. 8:**
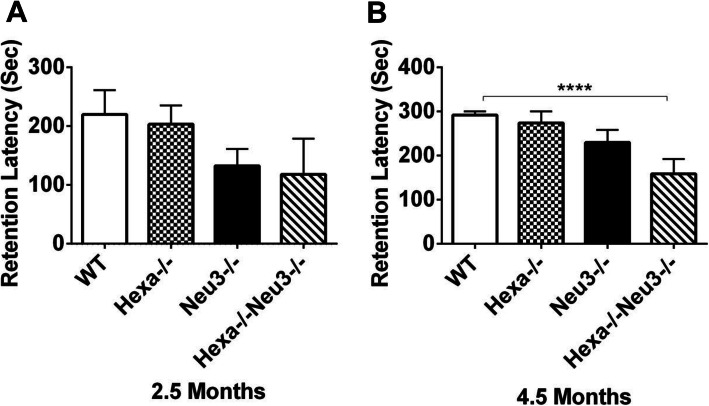
The passive-avoidance test. Latencies to enter the dark compartment on the 3rd day were shown for 2.5- (**a**) and 4.5-month-old (**b**) mice group. If mice did not enter the dark compartment within 300 s, retention latency time was recorded as 300 s. The data are presented as the mean ± SEM. One-way ANOVA was used for statistical analysis (****p < 0.001). A 2.5-month-old *WT* (*n* = 4), *Hexa*−/− (*n* = 6), *Neu3*−/− (*n* = 15), and *Hexa*−/−*Neu3*−/− (*n* = 6) mice; and 4.5-month-old *WT* (*n* = 10), *Hexa*−/− (*n* = 6), *Neu3*−/− (*n* = 10), and *Hexa*−/−*Neu3*−/− (*n* = 6)

The passive avoidance test was used to detect cognitive learning and memory deficits [43]. We found that 2.5-month-old *WT*, *Hexa*−/−, *Neu3*−/−, and *Hexa*−/−*Neu3*−/− mice re-entered the dark compartment at the average times: 219 ± 41.3 s, 203 ± 31.8 s, 132 ± 21.8 s, and 118 ± 60 s, respectively, after having previously received the electric shock through the feet. These results may indicate that the younger age group could not tolerate light, and prefer the dark compartment in spite of the 0.2 mA electric shock, for 2 s (Fig. 8a). Nearly all the 4.5-month-old *WT* mice avoided entrance into the dark compartment during the 300 s observation period, whereas the *Hexa*−/−*Neu3*−/− mice re-entered the chamber at an average time of 160 s (292.1 ± 7.9 s in *WT* and 159 ± 33.5 s in *Hexa*−/−*Neu3*−/−; *p* < 0.001). In addition, Hexa−/− and Neu3−/− mice re-entered the chamber in slightly less time than *WT* mice (Fig. 8b). These results may show that *Hexa*−/−*Neu3*−/− mice display a significant deterioration in memory function compared with that in other groups.

### Deficits in neuromotor behavior

**Fig. 9:**
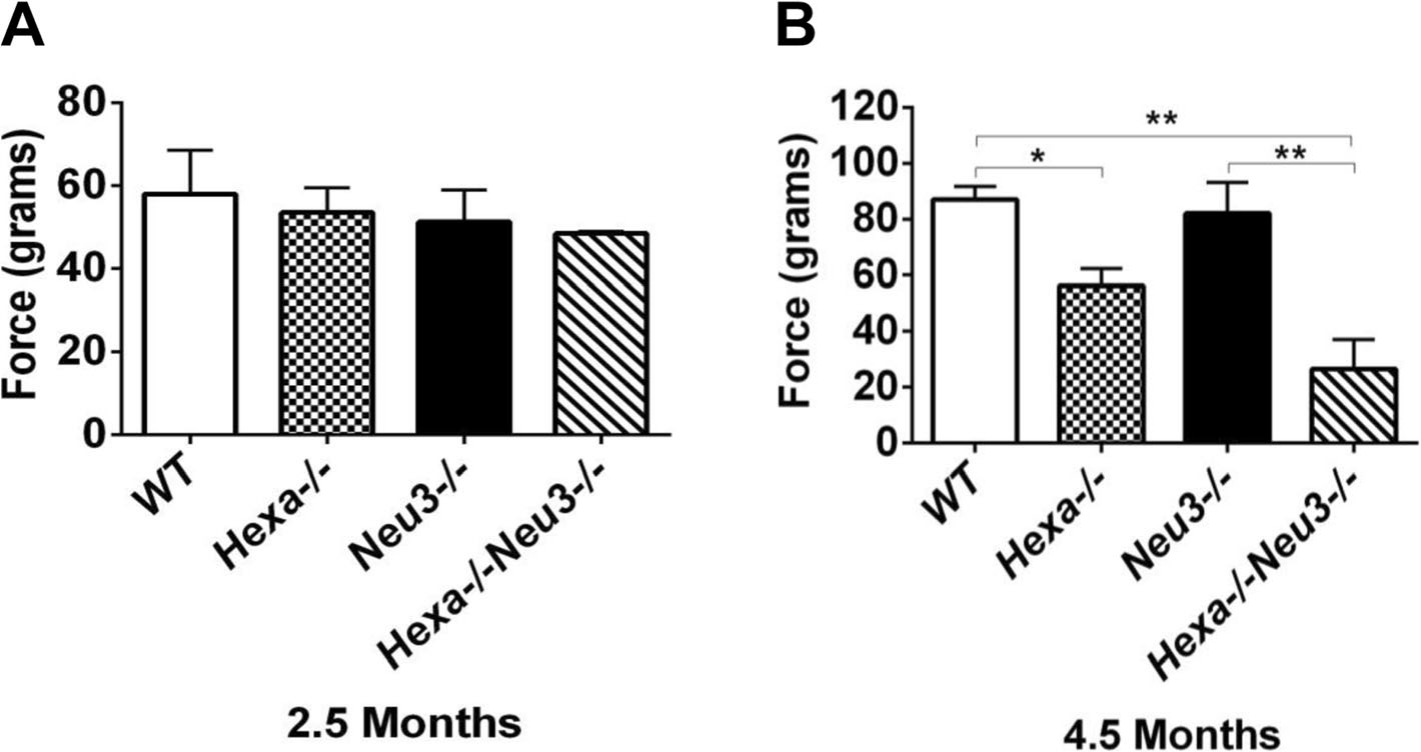
Forelimb grip strength measurement of 2.5- (**a**) and 4.5-month-old (**b**) mice group. The data are presented as the mean ± SEM. One-way ANOVA was used for statistical analysis (*p < 0.05, **p < 0.025). A 2.5-month-old *WT* (*n* = 5), *Hexa*−/− (*n* = 5), *Neu3*−/− (*n* = 4), and *Hexa*−/−*Neu3*−/− (*n* = 4) mice; 4.5-month-old *WT* (*n* = 4), *Hexa*−/− (*n* = 4), *Neu3*−/− (*n* = 4), and *Hexa*−/−*Neu3*−/− (*n* = 4) mice

In previous work, *Hexa*−/−*Neu3*−/− mice exhibited progressively impaired performance on the rotarod test [5]. This test is used to evaluate motor skill learning and involves endurance, motor coordination, and muscle strength [47]. The Morris water maze test showed that both 2.5- and 4.5-month-old *Hexa*−/−*Neu3*−/− mice lost their swimming speed, which could be related with deterioration in motor coordination and muscle strength (Fig. 7e, f). For a more specific measure of muscle strength, grip strength tests were also performed [44, 48]. The forelimb assessments demonstrated no significant change in strength of the 2.5-month-old mice (Fig. 9a). The strength of 4.5-month-old Hexa−/− and Hexa−/−Neu3−/− mice was significantly impaired compared to *WT* mice (87 ± 4.7 g in *WT*, 56.2 ± 6.1 g in Hexa−/−, 82 ± 11.2 g in Neu3−/−, and 26.5 ± 10.5 g in Hexa−/−Neu3−/−). Overall, *Hexa*−/−*Neu3*−/− mice displayed the most dramatic muscle strength impairment and functional deterioration, *p* < 0.025. This result could be related to abnormal GM2 accumulation in the muscles of *Hexa*−/−*Neu3*−/− mice [5].

## Discussion

Neuroinflammation is a common hallmark in the pathogenesis of several lysosomal storage disorders including GM2 gangliosidosis, and may negatively impact neuronal survival [17, 19]. In a previous study, we showed that severe neuronal apoptosis and astrogliosis in the hippocampus, cortex, and thalamus of *Hexa*−/−*Neu3*−/− mice correlated with undegraded GM2 ganglioside accumulation [5]. However, the mechanism of disease progression relevant to neuroinflammation was not yet elucidated. In the present work, we focused on investigating the age-dependent mediators of neuroinflammation in *Hexa*−/−*Neu3*−/− mice for future therapeutic studies.

It has been shown that the accumulation of undegraded gangliosides is linked to activation of microglial cells [30, 17]. Here, we demonstrated that an accumulation of GM2 ganglioside led to the activation of the microglial/macrophage system in the brain and retina of *Hexa*−/−*Neu3*−/− mice, compared with that in age-matched *WT*, *Hexa*−/−, and *Neu3*−/− mice. Activation of this cellular system led to an altered expression profile of both pro- and anti-inflammatory cytokines, as well as chemokines, in the cortex and cerebellum. We found that while pro-inflammatory cytokines and chemokines, such as Ccl2, Ccl3, Ccl4, and Cxcl10 were significantly upregulated in the brain of *Hexa*−/−*Neu3*−/− compared with that in *Hexa*−/− mice, anti-inflammatory cytokines and chemokines such as IL10, IL13, IL11, and IL24 were significantly downregulated (Fig. 1). It has been shown that the simultaneous increase of Ccl2, Ccl3, and Ccl5 in mice brain leads to epileptic seizures. Importantly, their elevated levels may account for the neuroinflammation and the seizure activity observed in the neuropathology of the early onset Tay-Sachs disease mouse model. Furthermore, these cytokines play a role in the attraction, migration, and activation of monocytes, lymphocytes, and neutrophils in the CNS [18, 24, 25]. Their increased levels in the CNS of *Hexa*−/−*Neu3*−/− mice triggered PBMC infiltration similar to the Sandhoff mouse model. This may suggest that inhibition of PBMC infiltration may delay disease progression and neurodegeneration in *Hexa*−/−*Neu3*−/− mice, similar to the *Hexb*−/−*Ccl2*−/− mice [36]. Moreover, it was reported that the deletion of the *Ccl3* gene caused a delay of neuronal loss in Sandhoff mice (*Hexb*−/−*Mip1α*−/− mice) by inhibiting macrophage infiltration to the inflammatory sites, which resulted in an improved neurologic status and a longer lifespan [37]. Additionally, *Hexb*−/−*Tnfα*−/− mice displayed a delayed neurodegenerative cascade by an extending lifespan, improving sensorimotor coordination, decreasing levels of astrogliosis and neuronal cell death [49]. We suggest that antagonists of pro-inflammatory cytokines and chemokines such as Ccl3 or Ccl4 may dampen neuroinflammation and improve disease outcomes in *Hexa*−/−*Neu3*−/− mice.

Activated microglia produce various pro-inflammatory mediators, chemokines, and cytokines which negatively impact neurons and oligodendrocytes [39]. Here, we showed that *Hexa*−/−*Neu3*−/− mice exhibited a significant loss in neuronal density and oligodendrocytes. These results are consistent with the previous research indicating increased apoptotic cell death in the brain of *Hexa*−/−*Neu3*−/− mice [5]. Increased level of Lif under inflammatory conditions has been shown to inhibit cell proliferation [50], a similar increase was observed in the cortex of *Hexa*−/−*Neu3*−/− mice. Furthermore, transcription levels of IL 11, whose overexpression has been shown to inhibit demyelinization by protecting oligodendrocytes [51], were significantly reduced in both the cortex and cerebellum of *Hexa*−/−*Neu3*−/− mice compared with that in *Hexa*−/− mice.

The Bmp protein family is responsible for the regulation of bone formation, maintenance, and repair [52]. Relatively low expression of Bmp 2, 4, 6, and 7 may be a contributing factor to the hunched posture observed in *Hexa*−/−*Neu3*−/− mice [5]. Furthermore, Csf3 is responsible for the production and differentiation of granulocytes and is involved in defense against pathogens. Relatively low expression of Csf3 might result in the predisposition of *Hexa*−/−*Neu3*−/− mice to bacterial infection which requires further investigation.

Deficiencies in memory, spatial and cognitive learning in Hexa−/−Neu3−/− mice, demonstrated by the Morris water maze and passive avoidance tests, might be related to abnormal GM2 accumulation in CNS as well as reduction in neuronal density and oligodendrocytes [30, 39]. Additionally, deficits in memory and learning might be related to impaired hippocampal function, which is involved in spatial and/or temporal processing of memory [53], deterioration of Purkinje cells, which play a role in spatial navigation [5, 54], and/or damaged retrosplenial and secondary motor cortices, which are involved in memory, navigation, and control of movement [55]. Memory impairments in *Hexa*−/−*Neu3*−/− mice, observed in the passive avoidance test, might also be accounted for by a decreased sensitivity to electric shock, due to damage in primary motor and somatosensory cortices, which are regions that process pain control [56].

The swimming speeds of 2.5- and 4.5-month-old *Hexa*−/−*Neu3*−/− mice were significantly lower than those of age-matched control groups, although they move equal distances to find the target platform (Fig. 7). These data suggest that Hexa−/−Neu3−/− mice may not be able to learn the location of the hidden platform due to the memory impairment and damaged signaling pathways in the CNS.

## Conclusion

In conclusion, our data suggest that abnormal GM2 ganglioside accumulation in the CNS of early onset Tay-Sachs disease mouse model activates neuroinflammation, by triggering the release of pro-inflammatory cytokines and chemokines, microgliosis, astrogliosis, and the infiltration of PBMC in the CNS. This inflammatory cascade results in a loss of neurons and oligodendrocytes. Our behavioral assessment identified abnormalities in behavior consistent with an important contribution of neuroinflammatory processes to subsequent neural dysfunctions. These observations collectively suggest that modulation of Ccl2, Ccl3, and Cxcl10 or of their receptors, as a potential therapeutic targets to slow down Tay-Sachs disease.

## Supplementary information

**Additional file 1: **Figure S1. Immunohistochemical analysis to detect microglial activation. The sections from the hippocampus (A, B, C, and D, respectively), cortex (E, F, G, and H, respectively), thalamus (I, J, K, and L, respectively), cerebellum (M, N, O and P, respectively) and pons (R, S, T, and U, respectively) of 2.5-month-old *WT*, *Hexa*-/-, *Neu3*-/- and *Hexa*-/- *Neu3*-/- mice were labeled with anti-Moma2 antibody (red), anti-lamp1 (green) and DAPI (blue). A yellow signal signifies the colocalization of Moma2 and lamp1 as an active microglial cell. The histograms represent the quantification of activated microglial cells in the hippocampus (V), cortex (W) thalamus (X) cerebellum (Y) and pons (Z). Scale bar = 50 μm. The data are represented as the mean ± S.E.M. One-way ANOVA was used for statistical analysis. (*p<0.05, **p<0.025, ***p<0.01 and ****p<0.001)

**Additional file 2: **Figure S2 Immunohistochemical analysis to detect microglial activation. The sections from the hippocampus, (A, B, C, and D, respectively), cortex (E, F, G, and H, respectively), thalamus (I, J, K, and L, respectively), cerebellum (M, N, O and P, respectively) and pons (Q, R, S and T, respectively) of 4.5-month-old *WT*, *Hexa*-/-, *Neu3*-/- and *Hexa*-/-*Neu3*- /- mice were stained with anti-Moma2 antibody (red), anti-Iba1 (green) and DAPI (blue). The yellow signal signifies the colocalization of Moma2 and Iba1 as phagocytic microglial cells. The histograms represent the quantification of Iba1 positive cells in the hippocampus (U), cortex (V), thalamus (W), cerebellum (X), and pons (Y). In the Hexa-/-Neu3-/- mice, colocalization of Moma2 with Iba1 (Z) was detected with ImageJ as percentage. Scale bar = 50 μm. The data are represented as the mean ± S.E.M. One-way ANOVA was used for statistical analysis. (*p<0.05, **p<0.025, ***p<0.01 and ****p<0.001)

**Additional file 3: **Figure S3. Immunohistochemical analysis of astrocytes. Sections from the hippocampus (A, B, C, and D, respectively), cortex (E, F, G, and H, respectively) and cerebellum (I, J, K, and L, respectively) of 2.5- and 4.5-month-old *WT* and *Hexa*-/-*Neu3*-/- mice were labeled with anti-GFAP antibody (red) and DAPI (blue) to detect astrogliosis. Scale bar = 50 μm.

**Additional file 4: **Figure S4. Immunohistochemical analysis to detect CD45+/Iba+ cells. The sections from the hippocampus, (A, B, C, and D, respectively), cortex (E, F, G, and H, respectively), thalamus (I, J, K, and L, respectively), cerebellum (M, N, O and P, respectively) and pons (Q, R, S and T, respectively) of 4.5-month-old *WT*, *Hexa*-/-, *Neu3*-/- and *Hexa*-/-*Neu3*- /- mice were stained with anti-CD45+ (red), anti-Iba1 (green) and DAPI (blue). The yellow signal signifies the colocalization of CD45 and Iba1 as microglial cells. In the *Hexa*-/-*Neu3*-/- mice, colocalization of CD45 with Iba1 (V) was detected with ImageJ as percentage. Scale bar = 50 μm. The data are represented as the mean ± S.E.M. One-way ANOVA was used for statistical analysis. (*p<0.05, **p<0.025, ***p<0.01 and ****p<0.001)

**Additional file 5: **Figure S5. Neuronal density detection for 2.5-month-old mice. The sections from the cortex (A, B, C, and D, respectively), thalamus (E, F, G, and H, respectively), cerebellum (I, J, K, and L, respectively) and pons (M, N, O and P, respectively) of 2.5-monthold *WT*, *Hexa*-/-, *Neu3*-/- and *Hexa*-/-*Neu3*-/- mice were labeled with anti-NeuN antibody (red), DAPI (blue). The histograms represent quantification of neuronal density for cortex (R), thalamus (S), cerebellum (T) pons (U). Scale bar = 50 μm for cortex and thalamus; 100 μm for cerebellum and pons. The data are represented as the mean ± S.E.M. One-way ANOVA was used for statistical analysis.

**Additional file 6: **Figure S6. Immunohistochemical staining for oligodendrocytes. The sections from the cortex (A, B, C, and D, respectively), thalamus (E, F, G, and H, respectively), cerebellum (I, J, K, and L, respectively) and pons (M, N, O and P, respectively) of 2.5-monthold *WT*, *Hexa*-/-, *Neu3*-/- and *Hexa*-/-*Neu3*-/- mice were labeled with anti-CNPase antibody (green) and DAPI (blue). The histograms represent the quantification of oligodendrocytes in the hippocampus (V), cortex (W), thalamus (X), cerebellum (Y) and pons (Z). Scale bar = 50 μm for cortex and thalamus; 100 μm for cerebellum and pons. The data are represented as the mean ± S.E.M. One-way ANOVA was used for statistical analysis.

**Additional file 7: **Figure S7. Immunohistochemistry analysis for IL-6 cytokine. The sections from the hippocampus (A, B, C, and D, respectively), cortex (E, F, G, and H, respectively), thalamus (I, J, K, and L, respectively), cerebellum (M, N, O and P, respectively) and pons (R, S, T, and U, respectively) of 4.5-months-old *WT*, *Hexa*-/-, *Neu3*-/- and *Hexa*-/-*Neu3*-/- mice were labeled with anti-IL6 antibody (red), and DAPI (blue). The histograms represent quantification of IL6 (+) cells in the hippocampus (V), cortex (W) thalamus (X) cerebellum (Y) and pons (Z). Scale bar = 50 μm. The data are represented as the mean ± S.E.M. One-way ANOVA was used for statistical analysis. (*p<0.05, **p<0.025, ***p<0.01 and ****p<0.001)

**Additional file 8: **Figure S8. Anxiety and locomotor activity were tested for 2.5- and 4.5-monthold *WT*, *Hexa*-/-, *Neu3*-/- and *Hexa*-/-*Neu3*-/- mice with the open field analysis. Time spent in the periphery (A) and the center (B) of the open field area were analyzed. The data are represented as the mean ± SEM. Schematic drawings of the zones and representative traces of 2.5- (C) and 4.5-month-old (D) mouse movement during the test. Two-way ANOVA was used for statistical analysis. (*p<0.05, **p<0.025, ***p<0.01 and ****p<0.001). 2.5-month-old *WT* (n=8), *Hexa*-/- (n=8), *Neu3*-/- (n=9), and *Hexa*-/-*Neu3*-/- (n=13) mice; 4.5-month-old *WT* (n=23), *Hexa*-/- (n=10), *Neu3*-/- (n=15), and *Hexa*-/-*Neu3*-/- (n=17)

## Data Availability

All data generated or analyzed during this study are included in this published article and its supplementary information files.

## Abbreviations

Actb: Beta-actin
anti-CNPase: 2′,3′-cyclic nucleotide 3′ phosphodiesterase antibody
anti-LAMP1: Lysosomal-associated membrane protein 1 antibody
anti-Moma2: Monocyte and macrophage antibody
anti-NeuN: Neuronal marker antibody
BBB: Blood–brain barrier
Bmp2: Bone morphogenetic protein 2
Bmp4: Bone morphogenetic protein 4
Bmp6: Bone morphogenetic protein 6
Bmp7: Bone morphogenetic protein 7
BSA: Bovine serum albumin
Csf1: Colony-stimulating factor 1
Csf3: Colony-stimulating factor 3
ENA-78: Epithelial neutrophil-activating protein 78
GADPH: D-glyceraldehyde-3-phosphate dehydrogenase
Gusb: Beta-glucuronidase
Hprt: Hypoxanthine-guanine phosphoribosyltransferase
Hsp90ab1: Heat shock protein 90 alpha family class B member
Iba1: Ionized calcium-binding adaptor molecule 1
IL1β: Interleukin 1 beta
IL2: Interleukin 2
IL4: Interleukin 4
IL6R: Interleukin-6 receptor
IL10: Interleukin 10
IL11: Interleukin 11
IL13: Interleukin 13
IL22: Interleukin 22
IP-10/Cxcl10: Interferon-inducible protein 10/chemokine interferon-γ inducible protein 10
KO: Knockout
Lif: Leukemia inhibitory factor
MAG: Myelin-associated glycoprotein
MBP: Myelin basic protein
MCP-1/Ccl2: Monocyte Chemoattractant Protein-1/C-C motif chemokine ligand 2
MIP-1α/Ccl3: Macrophage inflammatory protein 1 alpha/C-C motif chemokine ligand 3
MIP-1β/Ccl4: Macrophage ınflammatory protein 1 beta/C-C motif chemokine ligand 4
OCT: Optimal cutting temperature
PBMC: Peripheral blood mononuclear cell
PBS: Phosphate-buffered saline
PCR: Polymerase chain reaction
TGFβ1: Transforming growth factor beta 1
TNFα: Tumor necrosis factor-α
TNFR2: Tumor necrosis factor receptor-2
TSD: Tay-Sachs disease

## References

[1] Yamanaka S, Johnson MD, Grinberg A, Westphal H, Crawley JN, Taniike M (1994). Targeted disruption of the Hexa gene results in mice with biochemical and pathologic features of Tay-Sachs disease. Proc Natl Acad Sci U S A..

[2] Sango K, Yamanaka S, Hoffmann A, Okuda Y, Grinberg A, Westphal H (1995). Mouse models of Tay–Sachs and Sandhoff diseases differ in neurologic phenotype and ganglioside metabolism. Nat Genet..

[3] Phaneuf D, Wakamatsu N, Huang J, Borowski A, Peterson AC, Fortunato SR (1996). Dramatically different phenotypes in mouse models of human Tay-Sachs and Sandhoff diseases. Hum Mol Genet..

[4] Yuziuk JA, Bertoni C, Beccari T, Orlacchio A, Wu Y-Y, Li S-C, et al. Specificity of mouse G M2 activator protein and β-N-acetylhexosaminidases A and B. J Biol Chem. 1998;273:66–72. 10.1074/jbc.273.1.66 9417048

[5] Seyrantepe V, Demir SA, Timur ZK, Von Gerichten J, Marsching C, Erdemli E (2017). Murine sialidase Neu3 facilitates GM2 degradation and bypass in mouse model of Tay-Sachs disease. Exp Neurol..

[6] Jeyakumar M, Thomas R, Elliot-Smith E, Smitf DA, van der Spoel AC, D’Azzo A (2003). Central nervous system inflammation is a hallmark of pathogenesis in mouse models of GM1 and GM2 gangliosidosis. Brain..

[7] Wada R, Tifft CJ, Proia RL (2000). Microglial activation precedes acute neurodegeneration in Sandhoff disease and is suppressed by bone marrow transplantation. PNAS..

[8] Myerowitz R, Lawson D, Mizukami H, Mi Y, Tifft CJ, Proia RL (2002). Molecular pathophysiology in Tay-Sachs and Sandhoff diseases as revealed by gene expression profiling. Hum Mol Genet.

[9] Hayase T, Shimizu J, Goto T, Nozaki Y, Mori M, Takahashi N (2010). Unilaterally and rapidly progressing white matter lesion and elevated cytokines in a patient with Tay–Sachs disease. Brain Dev..

[10] Utz JRJ, Crutcher T, Schneider J, Sorgen P, Whitley CB (2015). Biomarkers of central nervous system inflammation in infantile and juvenile gangliosidoses. Mol Genet Metab..

[11] Baudry M, Yao Y, Simmons D, Liu J, Bi X (2003). Postnatal development of inflammation in a murine model of Niemann–Pick type C disease: immunohistochemical observations of microglia and astroglia. Exp Neurol..

[12] Farfel-Becker T, Vitner EB, Pressey SNR, Eilam R, Cooper JD, Futerman AH (2011). Spatial and temporal correlation between neuron loss and neuroinflammation in a mouse model of neuronopathic Gaucher disease. Hum Mol Genet..

[13] Wilkinson FL, Holley RJ, Langford-Smith KJ, Badrinath S, Liao A, Langford-Smith A (2012). Neuropathology in mouse models of mucopolysaccharidosis type I. IIIA and IIIB. PLoS One..

[14] Kollmann K, Uusi-Rauva K, Scifo E, Tyynelä J, Jalanko A, Braulke T (1832). Cell biology and function of neuronal ceroid lipofuscinosis-related proteins. Biochim Biophys Acta - Mol Basis Dis..

[15] Yamaguchi K, Shiozaki K, Moriya S, Koseki K, Wada T, Tateno H (2012). Reduced susceptibility to colitis-associated colon carcinogenesis in mice lacking plasma membrane-associated sialidase. PLoS One..

[16] Calhan OY, Seyrantepe V. Mice with catalytically inactive cathepsin a display neurobehavioral alterations. Behav Neurol. 2017;2017:1–11. 10.1155/2017/4261873 PMC5241486 28133419

[17] Vitner EB, Platt FM, Futerman AH (2010). Common and uncommon pathogenic cascades in lysosomal storage diseases. J Biol Chem..

[18] Reichel CA, Rehberg M, Lerchenberger M, Berberich N, Bihari P, Khandoga AG (2009). Ccl2 and Ccl3 mediate neutrophil recruitment via induction of protein synthesis and generation of lipid mediators. Arterioscler Thromb Vasc Biol..

[19] Bosch ME, Kielian T. Neuroinflammatory paradigms in lysosomal storage diseases. Front Neurosci. 2015;9:1–11. 10.3389/fnins.2015.00417 PMC4627351 26578874

[20] Arfi A, Richard M, Gandolphe C, Bonnefont-Rousselot D, Thérond P, Scherman D (2011). Neuroinflammatory and oxidative stress phenomena in MPS IIIA mouse model: the positive effect of long-term aspirin treatment. Mol Genet Metab..

[21] Villani lielmo RD, Gargiulo N, Faraonio R, Castaldo S, Reyero EG y, Di NP (2007). Cytokines, neurotrophins, and oxidative stress in brain disease from mucopolysaccharidosis IIIB. J Neurosci Res.

[22] Vitner EB, Farfel-Becker T, Eilam R, Biton I, Futerman AH (2012). Contribution of brain inflammation to neuronal cell death in neuronopathic forms of Gaucher’s disease. Brain..

[23] Pandey MK, Jabre NA, Xu YH, Zhang W, Setchell KDR, Grabowski GA (2014). Gaucher disease: chemotactic factors and immunological cell invasion in a mouse model. Mol Genet Metab..

[24] Mirones I, De Prada I, Gómez AM, Luque A, Martín R, Pérez-Jiménez MÁ (2013). A role for the CXCR3/CXCL10 axis in rasmussen encephalitis. Pediatr Neurol..

[25] Zhang X, Shen J, Man K, Chu ESH, Yau TO, Sung JCY (2014). CXCL10 plays a key role as an inflammatory mediator and a non-invasive biomarker of non-alcoholic steatohepatitis. J Hepatol..

[26] Zhang JM, An J (2007). Cytokines, inflammation, and pain. Int Anesthesiol Clin..

[27] Shah N, Kammermeier J, Elawad M, Glocker EO (2012). Interleukin-10 and interleukin-10-receptor defects in inflammatory bowel disease. Curr Allergy Asthma Rep..

[28] Bach JP, Rinn B, Meyer B, Dodel R, Bacher M (2008). Role of MIF in inflammation and tumorigenesis. Oncology..

[29] Pastore N, Brady OA, Diab HI, Martina JA, Sun L, Huynh T (2016). TFEB and TFE3 cooperate in the regulation of the innate immune response in activated macrophages. Autophagy..

[30] Rasband MN (2016). Glial contributions to neural function and disease. Mol Cell Proteomics..

[31] Ohmi K, Greenberg DS, Rajavel KS, Ryazantsev S, Li HH, Neufeld EF (2003). Activated microglia in cortex of mouse models of mucopolysaccharidoses I and IIIB. Proc Natl Acad Sci..

[32] Kavetsky L, Green KK, Boyle BR, Yousufzai FAK, Padron ZM, Melli SE, Kuhnel VL, Jackson HM, Blanco RE, Howell GR, Soto I. Increased interactions and engulfment of dendrites by microglia precede Purkinje cell degeneration in a mouse model of Niemann Pick Type-C. Sci Rep. 2019;9(1):14722. 10.1038/s41598-019-51246-1 PMC6788982 31605022

[33] Wu J, Yang S, Luo H, Zeng L, Ye L, Lu Y. Quantitative evaluation of monocyte transmigration into the brain following chemical opening of the blood–brain barrier in mice. Brain Res. 2006;1098(1):79–85. 10.1016/j.brainres.2006.04.074 PMC2830797 16908012

[34] Karperien A, Ahammer H, Jelinek HF (2013). Quantitating the subtleties of microglial morphology with fractal analysis. Front Cell Neurosci..

[35] da Fonseca ACC, Matias D, Garcia C, Amaral R, Geraldo LH, Freitas C, et al. The impact of microglial activation on blood-brain barrier in brain diseases. Front Cell Neurosci. 2014;8:1–13. 10.3389/fncel.2014.00362 PMC4217497 25404894

[36] Kyrkanides S, Miller AW, Miller JH, Tallents RH, Sabine M, Olschowka ME (2009). Peripheral blood mononuclear cell infiltration and neuroinflammation in the HexB-/- mouse model of neurodegeneration. J Neuroimmunol..

[37] Wu YP, Proia RL (2004). Deletion of macrophage-inflammatory protein 1α retards neurodegeneration in Sandhoff disease mice. Proc Natl Acad Sci U S A..

[38] Keilani S, Lun Y, Stevens AC, Williams HN, Sjoberg ER, Khanna R (2012). Lysosomal dysfunction in a mouse model of Sandhoff disease leads to accumulation of ganglioside-bound amyloid-␤ peptide. J Neurosci..

[39] Peferoen L, Kipp M, van der Valk P, van Noort JM, Amor S (2014). Oligodendrocyte-microglia cross-talk in the central nervous system. Immunology..

[40] Jackman N, Ishii A, Bansal R. Myelin biogenesis and oligodendrocyte development: parsing out the role of glycosphingolipids. Physiol. 2009;290–7. 10.1152/physiol.00016.2009 PMC2854184 19815855

[41] Vecino E, Rodriguez FD, Ruzafa N, Pereiro X, Sharma SC (2016). Glia-neuron interactions in the mammalian retina. Prog Retin Eye Res..

[42] Fu H, Cataldi MP, Ware TA, Zaraspe K, Meadows AS, Murrey DA, et al. Functional correction of neurological and somatic disorders at later stages of disease in MPS IIIA mice by systemic scAAV9-hSGSH gene delivery. Mol Ther - Methods Clin Dev. 2016;3:16036. 10.1038/mtm.2016.36 PMC4898406 27331076

[43] Hofmann L, Karl F, Sommer C, Üçeyler N (2017). Affective and cognitive behavior in the alpha-galactosidase A deficient mouse model of Fabry disease. PLoS One..

[44] D’Hooge R, Lüllmann-Rauch R, Beckers T, Balschun D, Schwake M, Reiss K (2005). Neurocognitive and psychotiform behavioral alterations and enhanced hippocampal long-term potentiation in transgenic mice displaying neuropathological features of human a-mannosidosis. J Neurosci..

[45] Schlegel V, Thieme M, Holzmann C, Witt M, Grittner U, Rolfs A (2016). Pharmacologic treatment assigned for Niemann Pick type C1 disease partly changes behavioral traits in wild-type mice. Int J Mol Sci..

[46] Seyrantepe V, Lema P, Caqueret A, Dridi L, Bel Hadj S, Carpentier S (2010). Mice doubly-deficient in lysosomal hexosaminidase A and neuraminidase 4 show epileptic crises and rapid neuronal loss. PLoS Genet..

[47] Foley JW, Bercury SD, Finn P, Cheng SH, Scheule RK, Ziegler RJ (2010). Evaluation of systemic follistatin as an adjuvant to stimulate muscle repair and improve motor function in Pompe mice. Mol Ther..

[48] Arisi GM, Foresti ML, Katki K, Shapiro LA. Increased CCL2, CCL3, CCL5, and IL-1β cytokine concentration in piriform cortex, hippocampus, and neocortex after pilocarpine-induced seizures. J Neuroinflammation. 2015;12:129. 10.1186/s12974-015-0347-z PMC4509848 26133170

[49] Abo-ouf H, Hooper AWM, White EJ, Van Rensburg HJJ, Trigatti BL, Igdoura SA (2013). Deletion of tumor necrosis factor-a ameliorates neurodegeneration in Sandhoff disease mice. Hum Mol Genet..

[50] Yue X, Wu L, Hu W (2015). The regulation of leukemia inhibitory factor. Cancer cell Microenviron..

[51] Maheshwari A, Janssens K, Bogie J, Van Den Haute C, Struys T, Lambrichts I, et al. Local overexpression of interleukin-11 in the central nervous system limits demyelination and enhances remyelination. Mediators Inflamm. 2013;2013. 10.1155/2013/685317 PMC3683504 23818742

[52] Sykaras N, Opperman LA (2003). Bone morphogenetic proteins (BMPs): how do they function and what can they offer the clinician?. J Oral Sci..

[53] Eichenbaum H (2017). On the integration of space, time, and memory. Neuron..

[54] Lee J-M, Kim C, Park J-M, Song M, Kim Y-J (2018). Effect of treadmill exercise on spatial navigation impairment associated with cerebellar Purkinje cell loss following chronic cerebral hypoperfusion. Mol Med Rep..

[55] Yamawaki N, Radulovic J, Shepherd GMG (2016). A corticocortical circuit directly links retrosplenial cortex to M2 in the mouse. J Neurosci..

[56] Lee S, Hwang E, Lee D, Choi JH (2017). Pulse-train stimulation of primary somatosensory cortex blocks pain perception in tail clip test. Exp Neurobiol..

