# Supplementary material for: GM2 ganglioside accumulation causes neuroinflammation and behavioral alterations in a mouse model of early onset Tay-Sachs disease
Source: J Neuroinflammation. 2020 Sep 20;17:277. doi: 10.1186/s12974-020-01947-6 (PMC7504627; doi:10.1186/s12974-020-01947-6)
Supplement: Supplementary file 1 — Additional file 1: Figure S1. Immunohistochemical analysis to detect microglial activation. The sections from the hippocampus (A, B, C, and D, respectively), cortex (E, F, G, and H, respectively), thalamus (I, J, K, and L, respectively), cerebellum (M, N, O and P, respectively) and pons (R, S, T, and U, respectively) of 2.5-month-old WT, Hexa-/-, Neu3-/- and Hexa-/- Neu3-/- mice were labeled with anti-Moma2 antibody (red), anti-lamp1 (green) and DAPI (blue). A yellow signal signifies the colocalization of Moma2 and lamp1 as an active microglial cell. The histograms represent the quantification of activated microglial cells in the hippocampus (V), cortex (W) thalamus (X) cerebellum (Y) and pons (Z). Scale bar = 50 μm. The data are represented as the mean ± S.E.M. One-way ANOVA was used for statistical analysis. (*p<0.05, **p<0.025, ***p<0.01 and ****p<0.001) [file 12974_2020_1947_MOESM1_ESM.pdf]

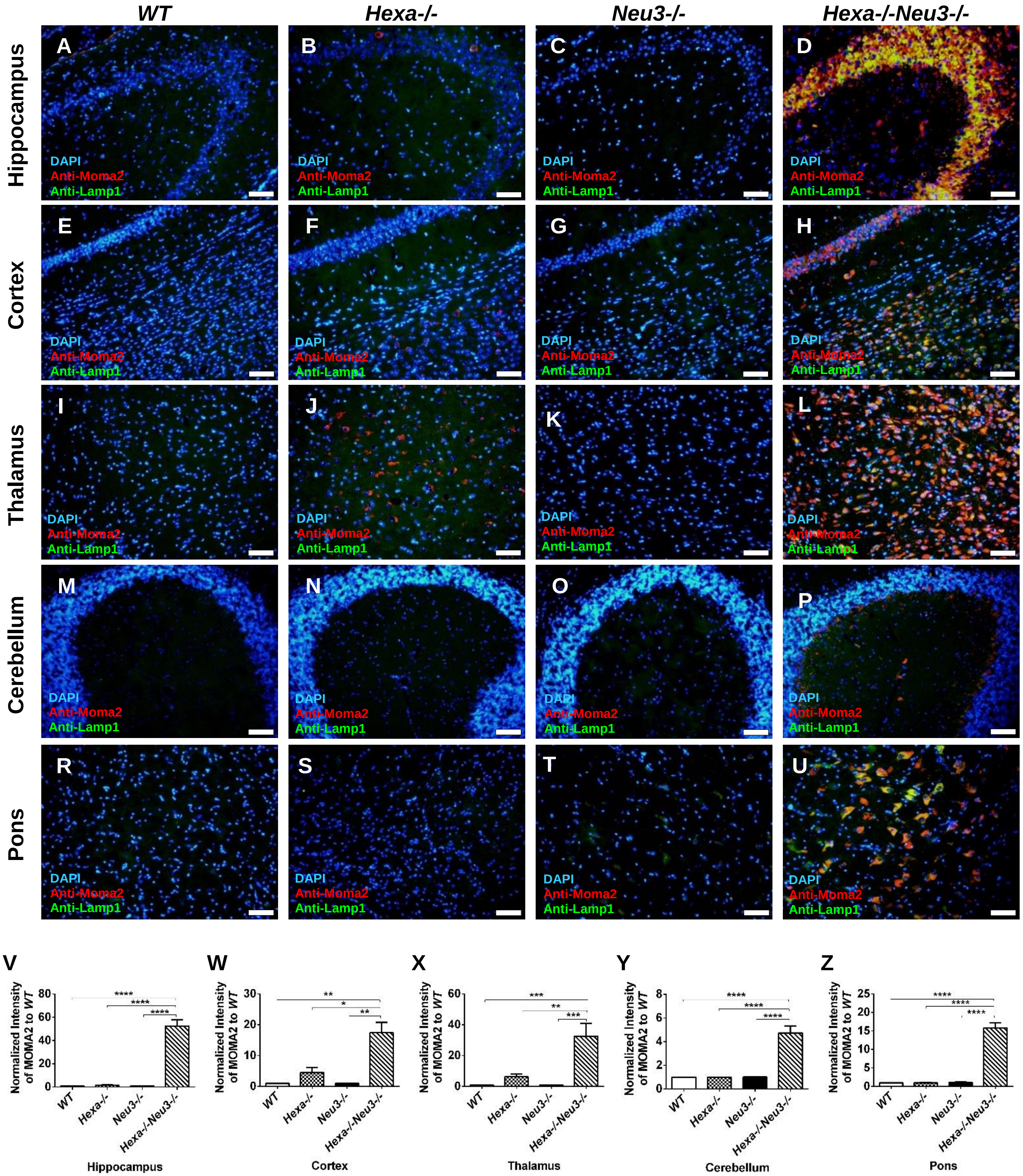

Supplementary Figure 1

Supplementary Figure 1. Immunohistochemical analysis to detect microglial activation. The sections from the hippocampus (A, B, C, and D, respectively), cortex (E, F, G, and H, respectively), thalamus (I, J, K, and L, respectively), cerebellum (M, N, O and P, respectively) and pons (R, S, T, and U, respectively) of 2.5-month-old *WT, Hexa*-/-, *Neu3*-/- and *Hexa*-/-*Neu3*-/- mice were labeled with anti-Moma2 antibody (red), anti-lamp1 (green) and DAPI (blue). A yellow signal signifies the colocalization of Moma2 and lamp1 as an active microglial cell. The histograms represent the quantification of activated microglial cells in the hippocampus (V), cortex (W) thalamus (X) cerebellum (Y) and pons (Z). Scale bar = 50 µm. The data are represented as the mean ± S.E.M. One-way ANOVA was used for statistical analysis. (*p<0.05, **p<0.025, ***p<0.01 and ****p<0.001)
