# Supplementary material for: GM2 ganglioside accumulation causes neuroinflammation and behavioral alterations in a mouse model of early onset Tay-Sachs disease
Source: J Neuroinflammation. 2020 Sep 20;17:277. doi: 10.1186/s12974-020-01947-6 (PMC7504627; doi:10.1186/s12974-020-01947-6)
Supplement: Supplementary file 2 — Additional file 2: Figure S2 Immunohistochemical analysis to detect microglial activation. The sections from the hippocampus, (A, B, C, and D, respectively), cortex (E, F, G, and H, respectively), thalamus (I, J, K, and L, respectively), cerebellum (M, N, O and P, respectively) and pons (Q, R, S and T, respectively) of 4.5-month-old WT, Hexa-/-, Neu3-/- and Hexa-/-Neu3- /- mice were stained with anti-Moma2 antibody (red), anti-Iba1 (green) and DAPI (blue). The yellow signal signifies the colocalization of Moma2 and Iba1 as phagocytic microglial cells. The histograms represent the quantification of Iba1 positive cells in the hippocampus (U), cortex (V), thalamus (W), cerebellum (X), and pons (Y). In the Hexa-/-Neu3-/- mice, colocalization of Moma2 with Iba1 (Z) was detected with ImageJ as percentage. Scale bar = 50 μm. The data are represented as the mean ± S.E.M. One-way ANOVA was used for statistical analysis. (*p<0.05, **p<0.025, ***p<0.01 and ****p<0.001) [file 12974_2020_1947_MOESM2_ESM.pdf]

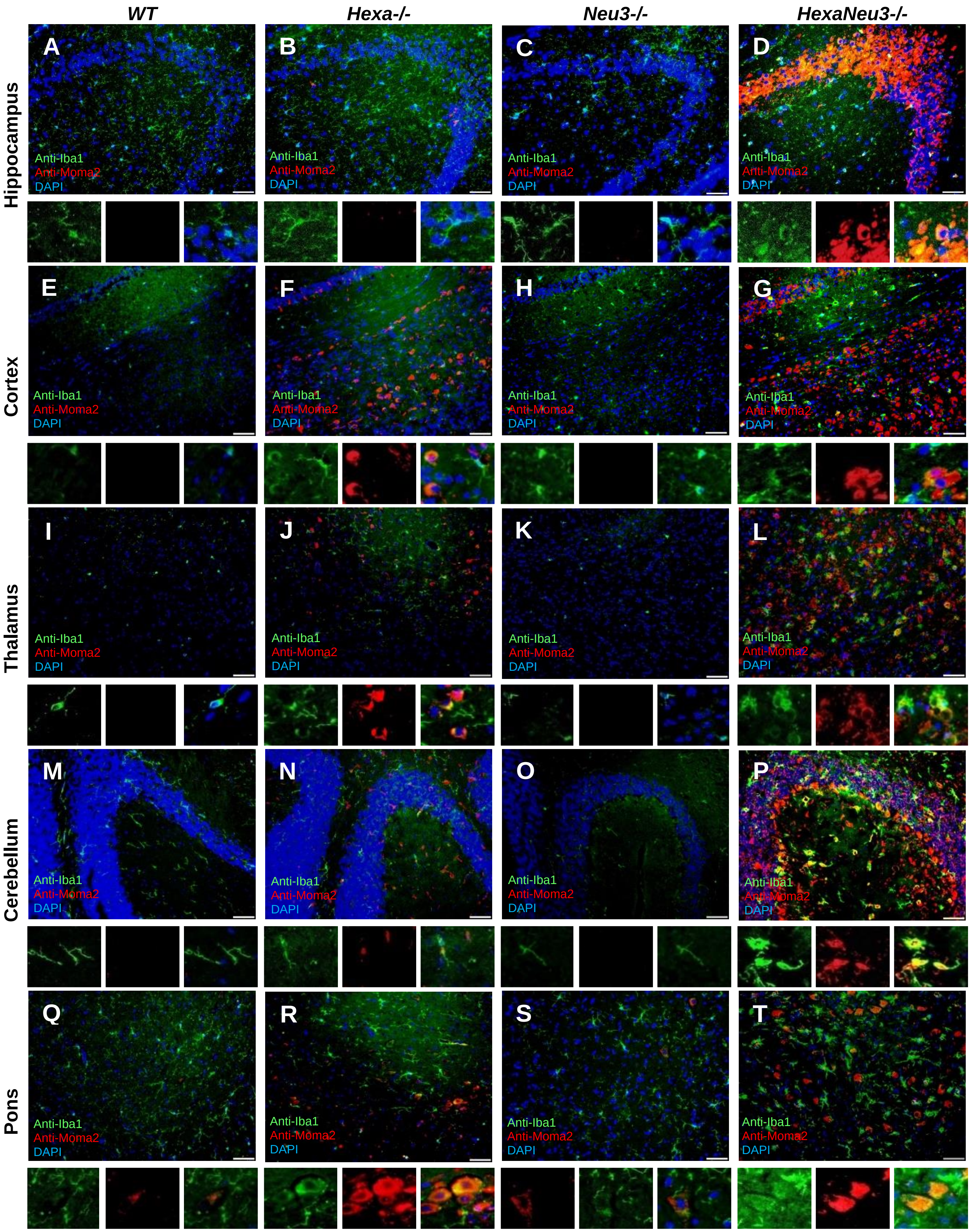

WT
Hexa-/-
Neu3-/-
HexaNeu3-/-
Hippocampus
Cortex
Thalamus
Cerebellum
Pons
A
B
C
D
E
F
H
G
I
J
K
L
M
N
O
P
Q
R
S
T
Anti-Iba1
Anti-Moma2
DAPI

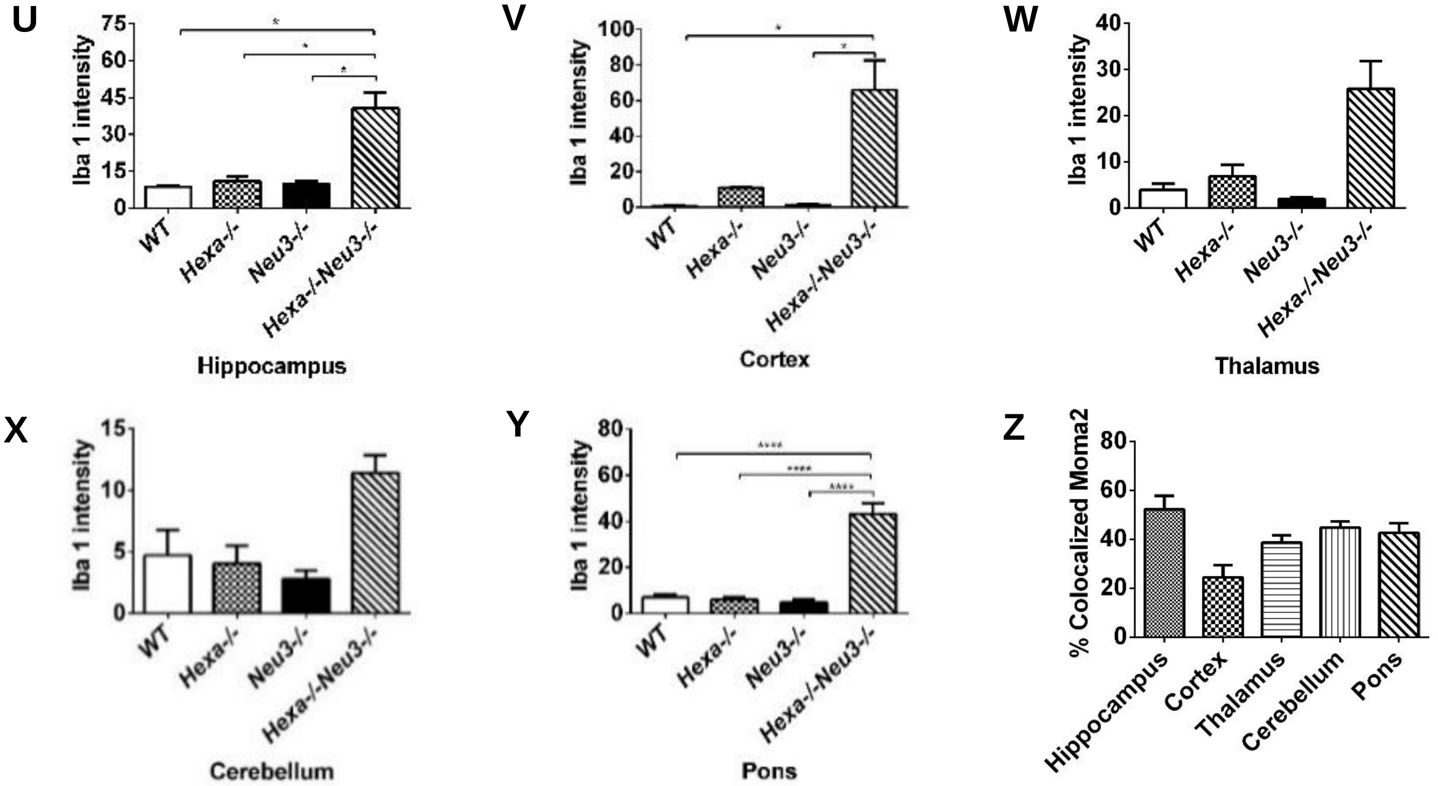

Supplementary Figure 2

Supplementary Figure 2. Immunohistochemical analysis to detect microglial activation. The sections from the hippocampus, (A, B, C, and D, respectively), cortex (E, F, G, and H, respectively), thalamus (I, J, K, and L, respectively), cerebellum (M, N, O and P, respectively) and pons (Q, R, S and T, respectively) of 4.5-month-old *WT*, *Hexa*-/-, *Neu3*-/- and *Hexa*-/-*Neu3*-/- mice were stained with anti-Moma2 antibody (red), anti-Iba1 (green) and DAPI (blue). The yellow signal signifies the colocalization of Moma2 and Iba1 as phagocytic microglial cells**.** The histograms represent the quantification of Iba1 positive cells in the hippocampus (U), cortex (V), thalamus(W), cerebellum (X), and pons (Y). In the *Hexa*-/-*Neu3*-/- mice, colocalization of Moma2 with Iba1 (Z) was detected with ImageJ as percentage. Scale bar = 50 µm. The data are represented as the mean ± S.E.M. One-way ANOVA was used for statistical analysis. (*$p<0.05$, **$p<0.025$, ***$p<0.01$ and ****$p<0.001$)
