# Supplementary material for: GM2 ganglioside accumulation causes neuroinflammation and behavioral alterations in a mouse model of early onset Tay-Sachs disease
Source: J Neuroinflammation. 2020 Sep 20;17:277. doi: 10.1186/s12974-020-01947-6 (PMC7504627; doi:10.1186/s12974-020-01947-6)
Supplement: Supplementary file 3 — Additional file 3: Figure S3. Immunohistochemical analysis of astrocytes. Sections from the hippocampus (A, B, C, and D, respectively), cortex (E, F, G, and H, respectively) and cerebellum (I, J, K, and L, respectively) of 2.5- and 4.5-month-old WT and Hexa-/-Neu3-/- mice were labeled with anti-GFAP antibody (red) and DAPI (blue) to detect astrogliosis. Scale bar = 50 μm. [file 12974_2020_1947_MOESM3_ESM.pdf]

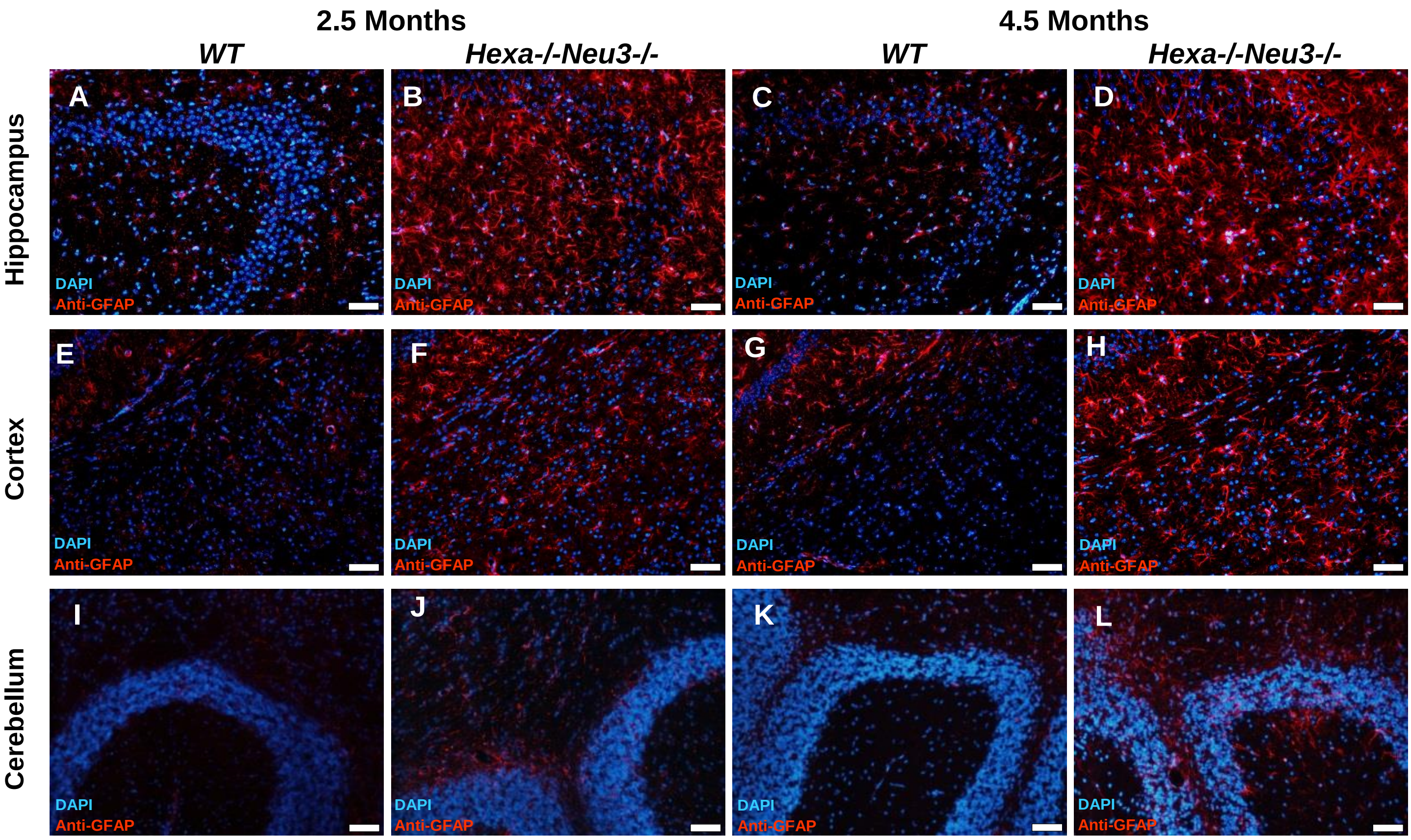

Supplementary Figure 3

Supplementary Figure 3. Immunohistochemical analysis of astrocytes. Sections from the hippocampus (A, B, C, and D, respectively), cortex (E, F, G, and H, respectively) and cerebellum (I, J, K, and L, respectively) of 2.5- and 4.5-month-old *WT* and *Hexa*-/-*Neu3*-/- mice were labeled with anti-GFAP antibody (red) and DAPI (blue) to detect astrogliosis. Scale bar = 50 μm.
