# Supplementary material for: GM2 ganglioside accumulation causes neuroinflammation and behavioral alterations in a mouse model of early onset Tay-Sachs disease
Source: J Neuroinflammation. 2020 Sep 20;17:277. doi: 10.1186/s12974-020-01947-6 (PMC7504627; doi:10.1186/s12974-020-01947-6)
Supplement: Supplementary file 4 — Additional file 4: Figure S4. Immunohistochemical analysis to detect CD45+/Iba+ cells. The sections from the hippocampus, (A, B, C, and D, respectively), cortex (E, F, G, and H, respectively), thalamus (I, J, K, and L, respectively), cerebellum (M, N, O and P, respectively) and pons (Q, R, S and T, respectively) of 4.5-month-old WT, Hexa-/-, Neu3-/- and Hexa-/-Neu3- /- mice were stained with anti-CD45+ (red), anti-Iba1 (green) and DAPI (blue). The yellow signal signifies the colocalization of CD45 and Iba1 as microglial cells. In the Hexa-/-Neu3-/- mice, colocalization of CD45 with Iba1 (V) was detected with ImageJ as percentage. Scale bar = 50 μm. The data are represented as the mean ± S.E.M. One-way ANOVA was used for statistical analysis. (*p<0.05, **p<0.025, ***p<0.01 and ****p<0.001) [file 12974_2020_1947_MOESM4_ESM.pdf]

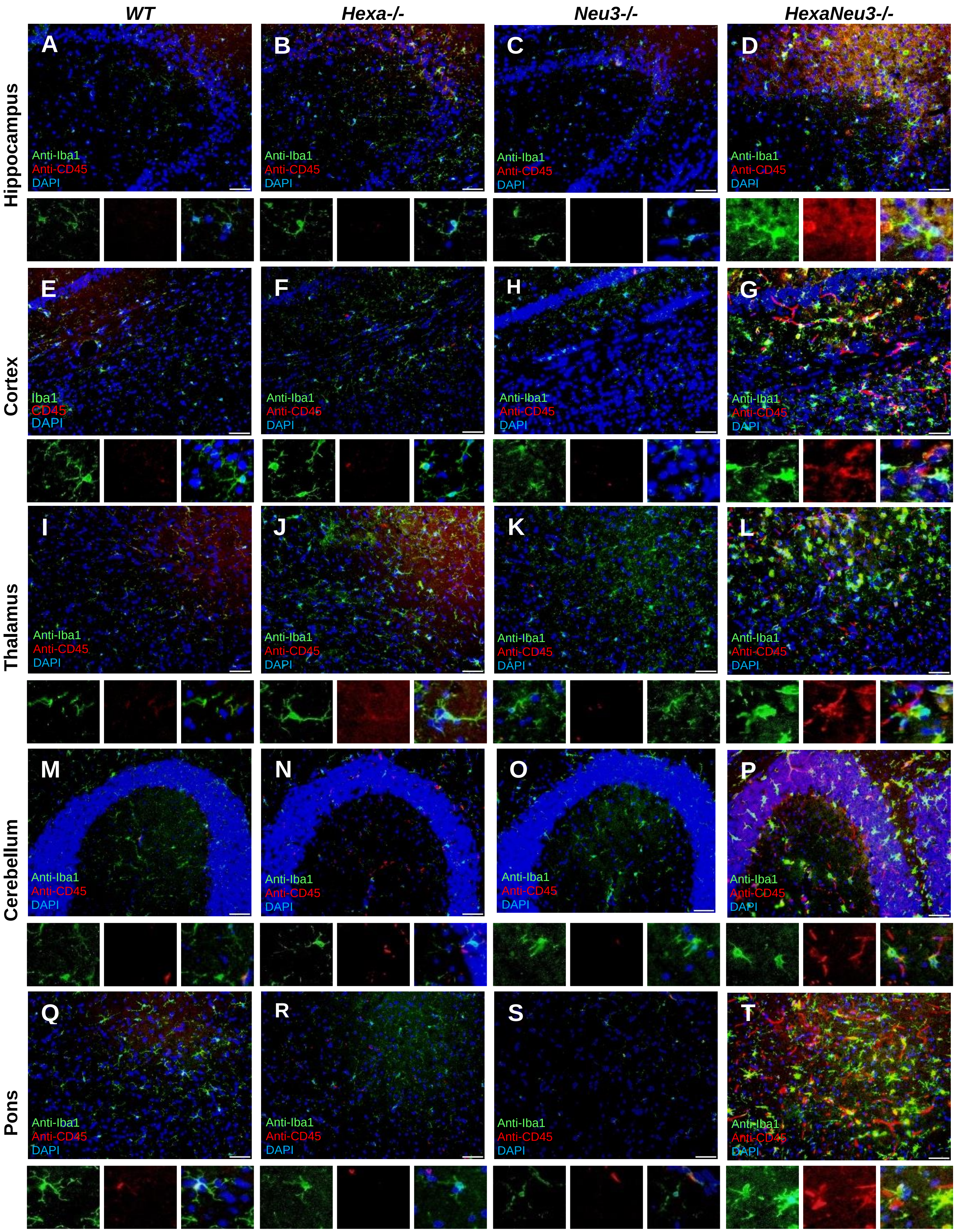
WT
Hexa-/-
Neu3-/-
HexaNeu3-/-
Hippocampus
Cortex
Thalamus
Cerebellum
Pons
A
B
C
D
E
F
H
G
I
J
K
L
M
N
O
P
Q
R
S
T
Anti-Iba1
Anti-CD45
DAPI
Iba1
CD45
DAPI

V

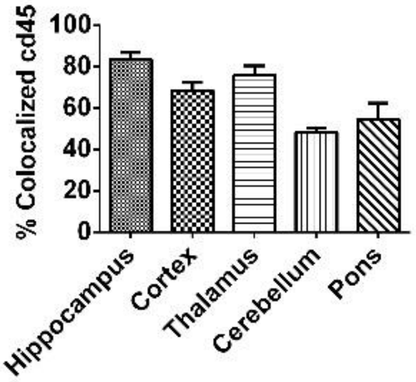

Supplementary Figure 4

Supplementary Figure 4. Immunohistochemical analysis to detect CD45+/Iba+ cells. The sections from the hippocampus, (A, B, C, and D, respectively), cortex (E, F, G, and H, respectively), thalamus (I, J, K, and L, respectively), cerebellum (M, N, O and P, respectively) and pons (Q, R, S and T, respectively) of 4.5-month-old *WT*, *Hexa*-/-, *Neu3*-/- and *Hexa*-/-*Neu3*-/- mice were stained with anti-CD45+ (red), anti-Iba1 (green) and DAPI (blue). The yellow signal signifies the colocalization of CD45 and Iba1 as microglial cells**.** In the *Hexa*-/-*Neu3*-/- mice, colocalization of CD45 with Iba1 (V) was detected with ImageJ as percentage. Scale bar = 50 μm. The data are represented as the mean ± S.E.M. One-way ANOVA was used for statistical analysis. (*p<0.05, **p<0.025, ***p<0.01 and ****p<0.001)
