# Supplementary material for: GM2 ganglioside accumulation causes neuroinflammation and behavioral alterations in a mouse model of early onset Tay-Sachs disease
Source: J Neuroinflammation. 2020 Sep 20;17:277. doi: 10.1186/s12974-020-01947-6 (PMC7504627; doi:10.1186/s12974-020-01947-6)
Supplement: Supplementary file 5 — Additional file 5: Figure S5. Neuronal density detection for 2.5-month-old mice. The sections from the cortex (A, B, C, and D, respectively), thalamus (E, F, G, and H, respectively), cerebellum (I, J, K, and L, respectively) and pons (M, N, O and P, respectively) of 2.5-monthold WT, Hexa-/-, Neu3-/- and Hexa-/-Neu3-/- mice were labeled with anti-NeuN antibody (red), DAPI (blue). The histograms represent quantification of neuronal density for cortex (R), thalamus (S), cerebellum (T) pons (U). Scale bar = 50 μm for cortex and thalamus; 100 μm for cerebellum and pons. The data are represented as the mean ± S.E.M. One-way ANOVA was used for statistical analysis. [file 12974_2020_1947_MOESM5_ESM.pdf]

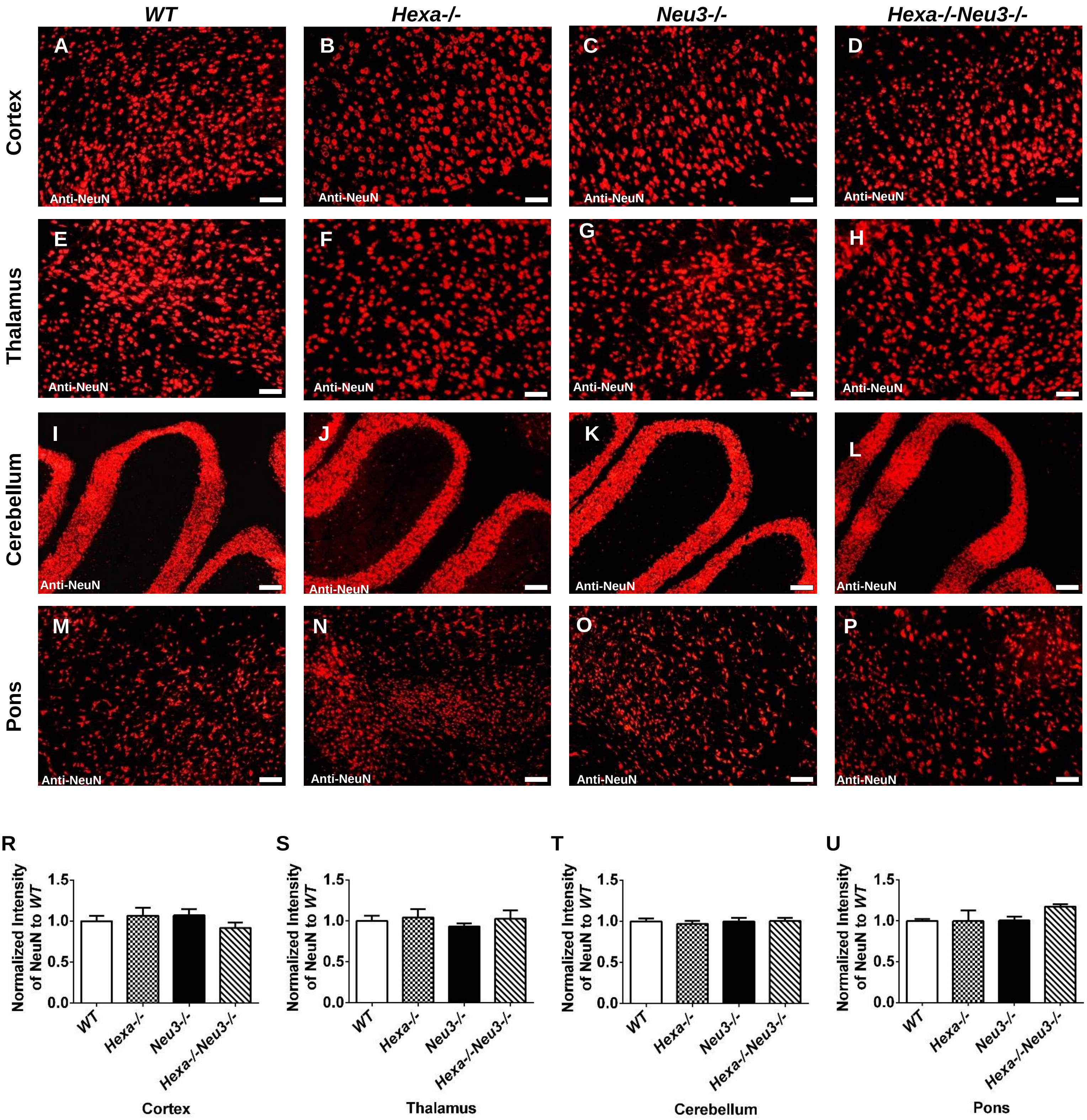

Supplementary Figure 5

Supplementary Figure 5. Neuronal density detection for 2.5-month-old mice. The sections from the cortex (A, B, C, and D, respectively), thalamus (E, F, G, and H, respectively), cerebellum (I, J, K, and L, respectively) and pons (M, N, O and P, respectively) of 2.5-month-old *WT, Hexa*-/-, *Neu3*-/- and *Hexa*-/-*Neu3*-/- mice were labeled with anti-NeuN antibody (red), DAPI (blue). The histograms represent quantification of neuronal density for cortex (R), thalamus (S), cerebellum (T) pons (U). Scale bar = 50 µm for cortex and thalamus; 100 µm for cerebellum and pons. The data are represented as the mean ± S.E.M. One-way ANOVA was used for statistical analysis.
