# Supplementary material for: GM2 ganglioside accumulation causes neuroinflammation and behavioral alterations in a mouse model of early onset Tay-Sachs disease
Source: J Neuroinflammation. 2020 Sep 20;17:277. doi: 10.1186/s12974-020-01947-6 (PMC7504627; doi:10.1186/s12974-020-01947-6)
Supplement: Supplementary file 6 — Additional file 6: Figure S6. Immunohistochemical staining for oligodendrocytes. The sections from the cortex (A, B, C, and D, respectively), thalamus (E, F, G, and H, respectively), cerebellum (I, J, K, and L, respectively) and pons (M, N, O and P, respectively) of 2.5-monthold WT, Hexa-/-, Neu3-/- and Hexa-/-Neu3-/- mice were labeled with anti-CNPase antibody (green) and DAPI (blue). The histograms represent the quantification of oligodendrocytes in the hippocampus (V), cortex (W), thalamus (X), cerebellum (Y) and pons (Z). Scale bar = 50 μm for cortex and thalamus; 100 μm for cerebellum and pons. The data are represented as the mean ± S.E.M. One-way ANOVA was used for statistical analysis. [file 12974_2020_1947_MOESM6_ESM.pdf]

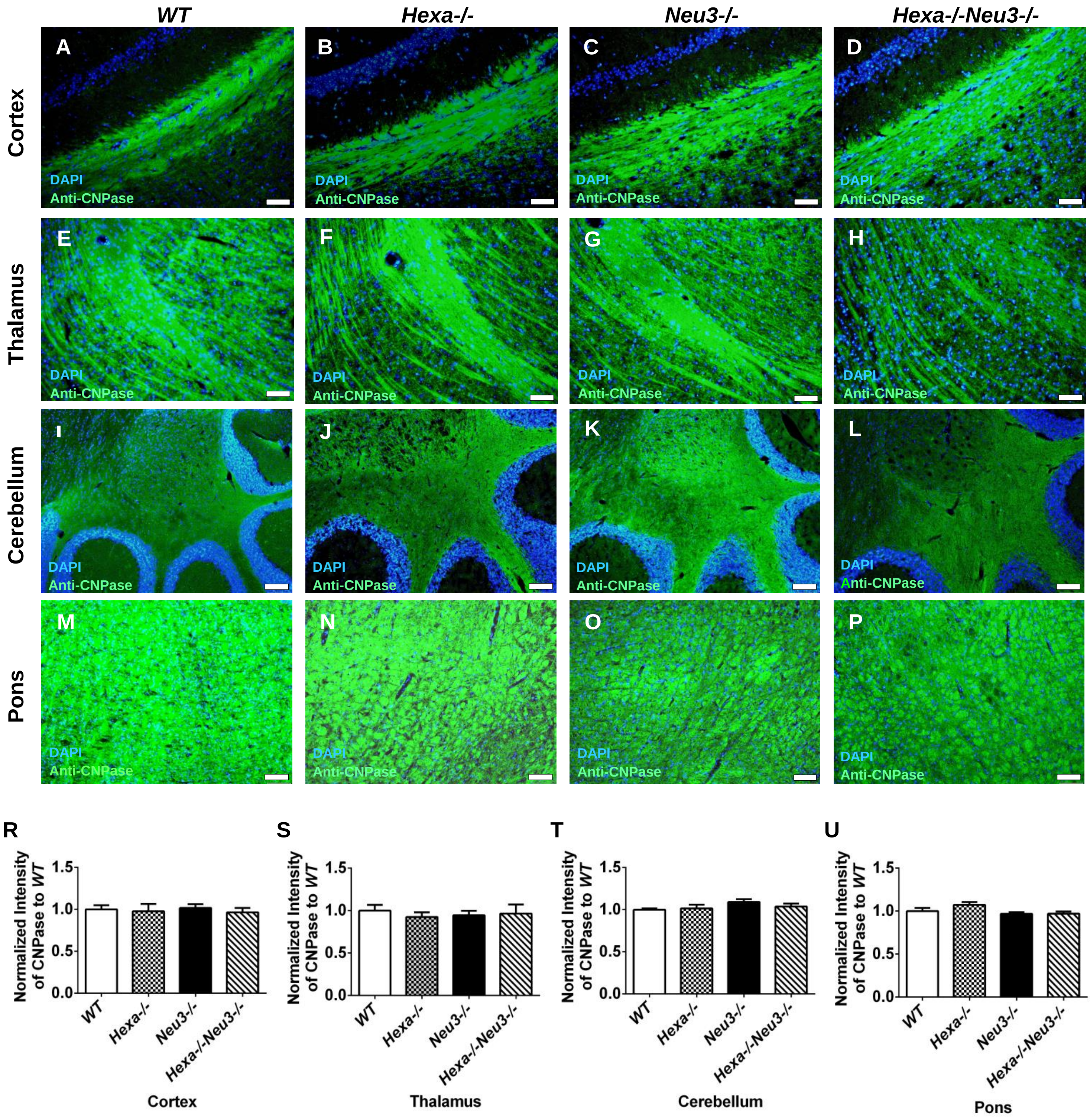

Supplementary Figure 6

Supplementary Figure 6. Immunohistochemical staining for oligodendrocytes. The sections from the cortex (A, B, C, and D, respectively), thalamus (E, F, G, and H, respectively), cerebellum (I, J, K, and L, respectively) and pons (M, N, O and P, respectively) of 2.5-month-old *WT, Hexa*-/-, *Neu3*-/- and *Hexa*-/-*Neu3*-/- mice were labeled with anti-CNPase antibody (green) and DAPI (blue). The histograms represent the quantification of oligodendrocytes in the hippocampus (V), cortex (W), thalamus (X), cerebellum (Y) and pons (Z). Scale bar = 50 µm for cortex and thalamus; 100 µm for cerebellum and pons. The data are represented as the mean ± S.E.M. One-way ANOVA was used for statistical analysis.
