# Supplementary material for: GM2 ganglioside accumulation causes neuroinflammation and behavioral alterations in a mouse model of early onset Tay-Sachs disease
Source: J Neuroinflammation. 2020 Sep 20;17:277. doi: 10.1186/s12974-020-01947-6 (PMC7504627; doi:10.1186/s12974-020-01947-6)
Supplement: Supplementary file 7 — Additional file 7: Figure S7. Immunohistochemistry analysis for IL-6 cytokine. The sections from the hippocampus (A, B, C, and D, respectively), cortex (E, F, G, and H, respectively), thalamus (I, J, K, and L, respectively), cerebellum (M, N, O and P, respectively) and pons (R, S, T, and U, respectively) of 4.5-months-old WT, Hexa-/-, Neu3-/- and Hexa-/-Neu3-/- mice were labeled with anti-IL6 antibody (red), and DAPI (blue). The histograms represent quantification of IL6 (+) cells in the hippocampus (V), cortex (W) thalamus (X) cerebellum (Y) and pons (Z). Scale bar = 50 μm. The data are represented as the mean ± S.E.M. One-way ANOVA was used for statistical analysis. (*p<0.05, **p<0.025, ***p<0.01 and ****p<0.001) [file 12974_2020_1947_MOESM7_ESM.pdf]

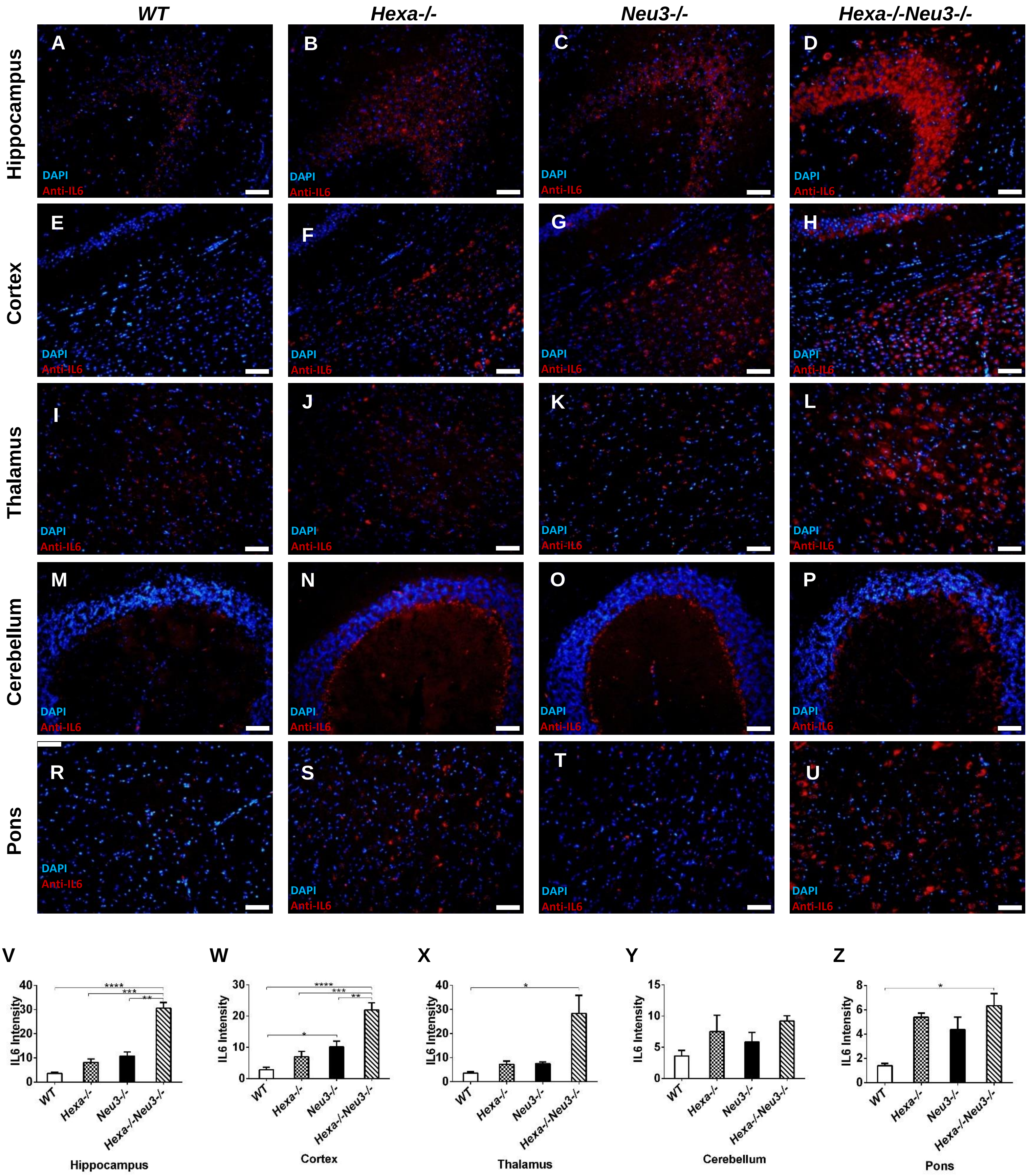

Supplementary Figure 7

Supplementary Figure 7. Immunohistochemistry analysis for IL-6 cytokine. The sections from the hippocampus (A, B, C, and D, respectively), cortex (E, F, G, and H, respectively), thalamus (I, J, K, and L, respectively), cerebellum (M, N, O and P, respectively) and pons (R, S, T, and U, respectively) of 4.5-months-old *WT, Hexa*-/-, *Neu3*-/- and *Hexa*-/-*Neu3*-/- mice were labeled with anti-IL6 antibody (red), and DAPI (blue). The histograms represent quantification of IL6 (+) cells in the hippocampus (V), cortex (W) thalamus (X) cerebellum (Y) and pons (Z). Scale bar = 50 μm. The data are represented as the mean ± S.E.M. One-way ANOVA was used for statistical analysis. (*$p<0.05$, **$p<0.025$, ***$p<0.01$ and ****$p<0.001$)
