# Supplementary material for: GM2 ganglioside accumulation causes neuroinflammation and behavioral alterations in a mouse model of early onset Tay-Sachs disease
Source: J Neuroinflammation. 2020 Sep 20;17:277. doi: 10.1186/s12974-020-01947-6 (PMC7504627; doi:10.1186/s12974-020-01947-6)
Supplement: Supplementary file 8 — Additional file 8: Figure S8. Anxiety and locomotor activity were tested for 2.5- and 4.5-monthold WT, Hexa-/-, Neu3-/- and Hexa-/-Neu3-/- mice with the open field analysis. Time spent in the periphery (A) and the center (B) of the open field area were analyzed. The data are represented as the mean ± SEM. Schematic drawings of the zones and representative traces of 2.5- (C) and 4.5-month-old (D) mouse movement during the test. Two-way ANOVA was used for statistical analysis. (*p<0.05, **p<0.025, ***p<0.01 and ****p<0.001). 2.5-month-old WT (n=8), Hexa-/- (n=8), Neu3-/- (n=9), and Hexa-/-Neu3-/- (n=13) mice; 4.5-month-old WT (n=23), Hexa-/- (n=10), Neu3-/- (n=15), and Hexa-/-Neu3-/- (n=17) [file 12974_2020_1947_MOESM8_ESM.pdf]

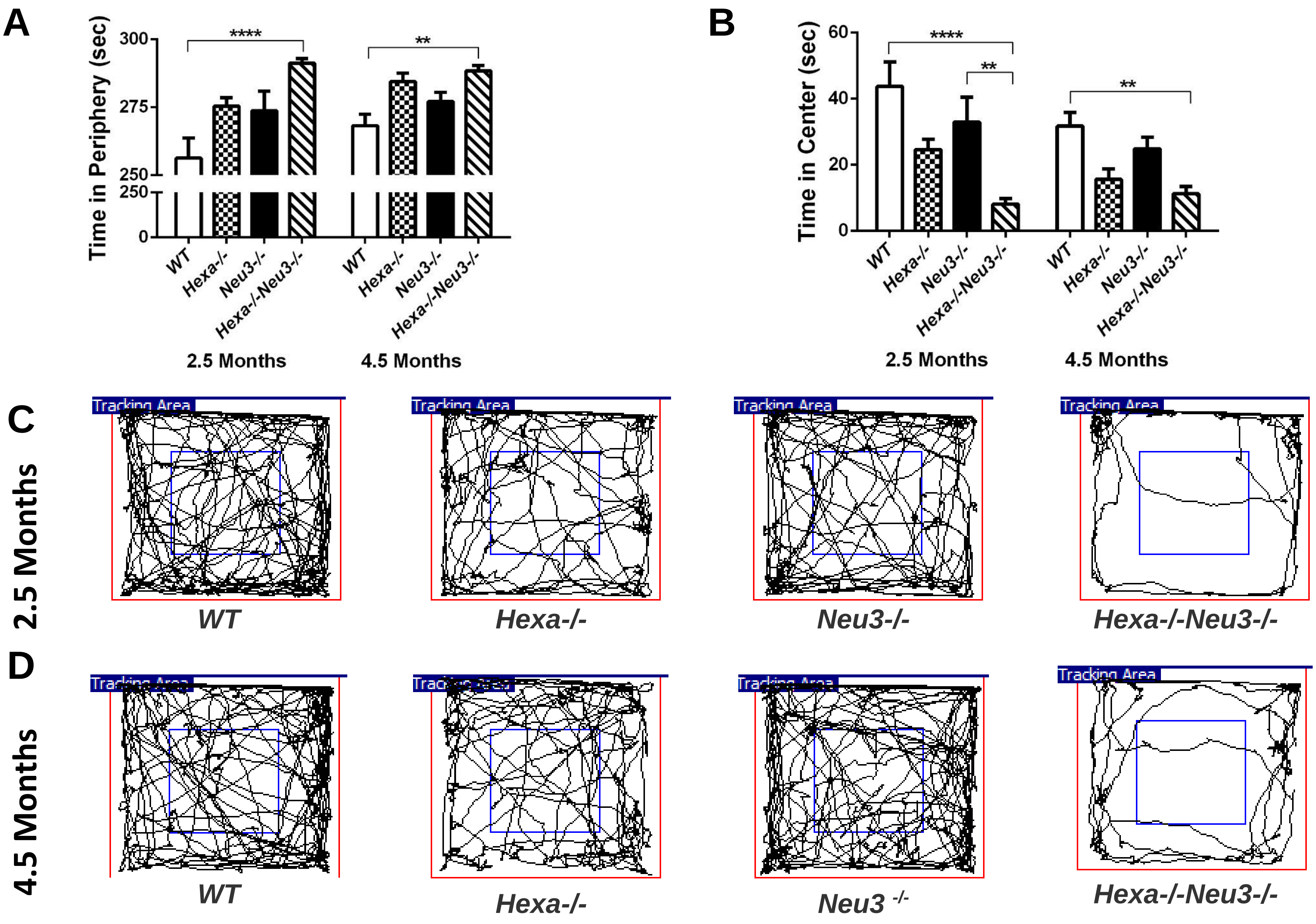

Supplementary Figure 8

Supplementary Figure 8. Anxiety and locomotor activity were tested for 2.5- and 4.5-month-old *WT*, *Hexa*-/-, *Neu3*-/- and *Hexa*-/-*Neu3*-/- mice with the open field analysis. Time spent in the periphery (A) and the center (B) of the open field area were analyzed. The data are represented as the mean ± SEM. Schematic drawings of the zones and representative traces of 2.5- (C) and 4.5-month-old (D) mouse movement during the test. Two-way ANOVA was used for statistical analysis. (*p<0.05, **p<0.025, ***p<0.01 and ****p<0.001). 2.5-month-old *WT* (n=8), *Hexa*-/- (n=8), *Neu3*-/- (n=9), and *Hexa*-/-*Neu3*-/- (n=13) mice; and 4.5- month-old *WT* (n=23), *Hexa*-/- (n=10), *Neu3*-/- (n=15), and *Hexa*-/-*Neu3*-/- (n=17)
